# Supplementary material for: Analysis of historical selection in winter wheat
Source: Theor Appl Genet. 2022 Jul 21;135(9):3005–23. doi: 10.1007/s00122-022-04163-3 (PMC9482581; doi:10.1007/s00122-022-04163-3)
Supplement: Supplementary file 1 — Supplementary file1 (PDF 3751 KB) [file 122_2022_4163_MOESM1_ESM.pdf]

Supplementary material for:

Analysis of historical selection in winter wheat

Chin Jian Yang*

Olufunmilayo Ladejobi†

Richard Mott†

Wayne Powell*

Ian Mackay*,‡

*Scotland's Rural College (SRUC), Kings Buildings, West Mains Road, Edinburgh, EH9 3JG, UK.

†Department of Genetics, Evolution & Environment, University College London, London, WC1E 6BT

‡IMplant Consultancy Ltd., Chelmsford, UK.

Supplementary Methods

Estimating multivariate selection parameters

We calculated the genetic variance-covariance (G) matrix for all 12 traits in the TG panel GBS marker data (Ladejobi et al. 2019). The variances were estimated from a univariate model (Equation 14) while the covariances were estimated from a bivariate model (Equation 15). All models were fitted using the *mmer* function from "sommer" (Covarrubias-Pazaran 2016) in R (R Core Team 2021). The model terms are similar to Equation 11 except that g follows a normal distribution of $N(0, \sigma_g^2 K)$ while g_1 and g_2 follow a multivariate normal distribution of $MVN(0, \begin{bmatrix} \sigma_{g1}^2 & \sigma_{g1,g2} \\ \sigma_{g1,g2} & \sigma_{g2}^2 \end{bmatrix} \otimes K)$. The diagonal terms σ_{g1}^2 and σ_{g2}^2 are the genetic variances for trait 1 and 2, and the off-diagonal term $\sigma_{g1,g2}$ is the genetic covariance between trait 1 and 2. The residual effect ε_1 and ε_2 follow a multivariate normal distribution of $MVN(0, \begin{bmatrix} \sigma_{\varepsilon1}^2 & \sigma_{\varepsilon1,\varepsilon2} \\ \sigma_{\varepsilon1,\varepsilon2} & \sigma_{\varepsilon2}^2 \end{bmatrix} \otimes I)$.

$$y = X\beta + Zg + \varepsilon \qquad \text{[Equation 14]}$$

$$\begin{cases} y_1 = X\beta_1 + Zg_1 + \varepsilon_1 \\ y_2 = X\beta_2 + Zg_2 + \varepsilon_2 \end{cases} \qquad \text{[Equation 15]}$$

Similar to above, we calculated the G-matrices for each country of origin (G_{DE}, G_{FR}, G_{UK}) and year group (G_{y1}, G_{y2}, G_{y3}) to test if G is stable across geographical and temporal variations in the TG panel. We divided the panel into three approximately equal year groups by choosing varieties in 1990 or earlier as group 1, after 1990 but before 2002 as group 2, and 2002 or later as group 3. We used Random Skewers (RS) (Cheverud and Marroig 2007) as provided by the *skewers* function from the "phytools" package (Revell 2012) in R (R Core Team 2021) to test if any two G's are different. Specifically, RS measures the correlations in their selection responses ΔZ when two G's are multiplied by random selection gradients β_{sel}. We tested if G_{DE}, G_{FR} and G_{UK} are different from each other and also if they are different from G. Similar comparisons were applied to G_{y1}, G_{y2}, and G_{y3}. We also repeated all of the comparisons using the Mantel test (Mantel 1967) as provided by the *mantel.test* function from the ape" package (Paradis et al. 2004) in R (R Core Team 2021).

In addition, we calculated the genetic correlation (g) matrices by scaling all G matrices by their diagonals (variances). We repeated all of the comparisons using RS and Mantel test in the g matrices. The use of g matrices eliminates any variation due to the differences in trait scales.

Next, we estimated the G_{DArT} and g_{DArT} from the TG panel DArT marker data (Bentley et al. 2014) as a check if G and other selection parameters are sensitive to the choice of marker data. To do so, we applied the same RS and Mantel test to check between G and G_{DArT} as well as between g and g_{DArT}.

As elucidated in the RS test, the multivariate breeder's equation of $\Delta Z = G\beta_{sel}$ (Lande and Arnold 1983) can be applied to estimate the selection gradient in the TG panel. Using a simple linear regression of trait j as the dependent variable and the variety year of release and country of origin as independent variables, we obtained the year regression coefficient as an estimate for the annual selection response ΔZ. Given ΔZ and G, we solved the multivariate breeder's equation and obtained the annual selection gradient β_{sel}. We also derived the phenotypic variance-covariance matrix (P) as a sum of genetic (G) and residual (E) variance-covariance matrix. This allowed us to solve the equation of $S = P\beta_{sel}$ and $i =$

$S/\sqrt{diag(P)}$ for the annual selection differentials S (Falconer and Mackay 1996) and annual selection intensity i.

Additionally, we decomposed the selection responses ΔZ and differentials S into direct and indirect components. This calculation is explained in the following equation for ΔZ, which can be easily translated for S and i.

$$\Delta Z = \begin{bmatrix} \Delta Z_1 \\ \Delta Z_2 \\ \vdots \\ \Delta Z_{12} \end{bmatrix} = G\beta_{sel} = \begin{bmatrix} G_{1,1} & G_{1,2} & \cdots & G_{1,12} \\ G_{2,1} & G_{2,2} & \vdots & G_{2,12} \\ \vdots & \cdots & \ddots & \vdots \\ G_{12,1} & G_{12,2} & \cdots & G_{12,12} \end{bmatrix} \begin{bmatrix} \beta_{sel,1} \\ \beta_{sel,2} \\ \vdots \\ \beta_{sel,12} \end{bmatrix} \quad \text{[Equation 16]}$$

Following Equation 16, trait 1 selection response can be written as $\Delta Z_1 = G_{1,1}\beta_{sel,1} + G_{1,2}\beta_{sel,2} + \cdots + G_{1,12}\beta_{sel,12}$. The direct response is $G_{1,1}\beta_{sel,1}$ due to selection gradient $\beta_{sel,1}$ acting on the genetic variance $G_{1,1}$, and the indirect response is $G_{1,2}\beta_{sel,2} + \cdots + G_{1,12}\beta_{sel,12}$ due to selection gradients on other traits acting on the genetic covariances. Therefore, the indirect response of trait j is caused by genetic correlations between trait j and other traits that are under selection. This explanation here can be extended to other traits, as well as the direct and indirect selection differentials.

Multivariate selection parameters in simulated example

We simulated a single generation of selection to investigate if it is possible to obtain correct selection parameters from the multivariate breeder's equation. All simulation was done using AlphaSimR (Gaynor et al. 2021). The simulation used the same fictitious species with 10 chromosomes as described previously. We created 10 unrelated founders and made 300 random crosses and retained 1 F_1 individual from each cross. We then created one doubled-haploid (DH) individual from each of the F_1 individual. These 300 DH individuals became the pre-selected population. We simulated three traits and 15 QTLs for each trait, where there are 9 unique QTLs for each trait, two pleiotropic QTLs that are shared by any two traits, and 2 pleiotropic QTLs that are shared by all three traits. This ensured that the traits have some degree of correlation. We set the QTL effects (a) based on the founder allele frequencies: one $4\mathrm{a}$-QTL at 0.1 frequency, two $3\mathrm{a}$-QTL at 0.2 frequency, four $2\mathrm{a}$-QTL at 0.3 frequency, and eight a-QTL at 0.4 to 0.5 frequency. We scaled all QTL effects such that the additive genetic variance is 1, and we added residual effect with a variance of 1 so that all traits have a heritability of 0.5.

Next, we applied three different selection schemes: select for individuals with top 10% of trait 1 (Sel1), select for individuals with either top 10% of trait 1 or 2 (Sel2), and select for individuals with top 10% of trait 1, 2 or 3 (Sel3). The differences between mean of selected individuals and mean of pre-selected population were calculated as the true selection differentials (S). Similar to previous crosses with the founders, we made 300 random crosses among the selected individuals and created a DH individual from each of the resulting F_1 individual. These 300 individuals became the post-selected population. The differences between mean of post- and pre-selected populations were calculated as the realized selection responses ($\Delta\mathrm{Z}$). We calculated the true genetic (G) and phenotypic (P) variance-covariance matrices directly from the genetic values in the pre-selected population, and we also estimated G and P from the pre-selected population using the mixed linear model approach as shown in Equation 14 and 15.

For Sel1, Sel2 and Sel3, we determined the true, realized and estimated multivariate selection parameters. We obtained the true β_{sel}, Z and i from solving $S = P\beta_{sel}$, $\Delta Z = G\beta_{sel}$ and $i = S/\sqrt{diag(P)}$ with true S, G and P. We calculated the realized β_{sel}, S and i from solving $\Delta Z = G\beta_{sel}$, $S = P\beta_{sel}$, and $i =$

$S/\sqrt{diag(P)}$ with realized ΔZ and true G and P. Lastly, we computed the realized β_{sel}, S and i from solving $\Delta Z = G\beta_{sel}$, $S = P\beta_{sel}$, and $i = S/\sqrt{diag(P)}$ with realized ΔZ and estimated G and P. We evaluated the accuracies of multivariate selection parameters by correlating the true values against either realized or estimated values.

Supplementary Results

Multivariate selection parameters in the TG panel

We calculated the genetic variance-covariance (G) matrix for all 12 traits using the TG panel GBS marker data (Table S11) as a pre-requisite to the multivariate selection analysis. To verify that G is representative across years of release and countries of origin, we compared the overall G with G's calculated from subsets of the TG panel for three year-groups of roughly equal number of varieties (G_{y1}: 1948 to 1990, G_{y2}: 1991 to 2001, G_{y3}: 2002 to 2007) and three countries of origin (G_{DE}: Germany, G_{FR}: France, G_{UK}: United Kingdom). The comparisons suggested that the G's are similar when tested using Random Skewers (Cheverud and Marroig 2007) but less so when tested using Mantel test (Mantel 1967) (Table S10). Because the G's may be influenced by the trait magnitudes, we also compared the genetic correlation (g) matrix similarly. In contrast to the previous comparisons with G's, the results for g's indicated that the matrices were more dissimilar when tested using Randoms Skewers but more similar when tested using Mantel test (Table S10). However, we found higher similarities between G and $G_{y1,y2,y3}$ or between G and $G_{DE,FR,UK}$ than among $G_{y1,y2,y3}$ or $G_{DE,FR,UK}$. This result indicates that the overall G is a reasonable average estimate of the variations that exist due to years of release and countries of origin.

Using the selection response (ΔZ) estimated from linear regression of each trait over years of release and G, we computed the selection gradient (β_{sel}) from the multivariate breeder's equation of $\Delta Z = G\beta_{sel}$ (Table S8). Between ΔZ and β_{sel}, the directions of the vectors' elements are only the same for 7 out of 12 traits. This suggests that the selection response is divergent from the selection gradient, which is the relationship between trait value and individual fitness (Lande and Arnold 1983). The divergence is almost perpendicular at angle of 85.8° between the two vectors, therefore implies that a strong selection gradient is required to achieve the desired selection response. In addition, we converted β_{sel} into an easier-to-understand measure called the selection differential (S) (Table S8) that is computed from $S = P\beta_{sel}$ where P is the phenotypic variance-covariance matrix. Similar to its univariate counterpart, S is the difference between the mean of selected subset from the mean of population. Like our previous results on ΔZ and β_{sel}, the directions of ΔZ and S elements are the same for only 5 out of 12 traits with an angle of 79.1° between the two vectors. We also computed the selection intensity (i) which is a standardized measure of S (Table S8). Overall, the selection force, as measured by β_{sel}, S and i, is not well aligned with the selection response, ΔZ.

Subsequently, we showed the partitioning of ΔZ, S and i into the direct and indirect components by isolating the products of β_{sel} with the matrix diagonals (direct) and matrix off-diagonals (Table S8). This method allows us to quantify the amount of each selection measure that is directly due to the available variation within a trait or indirectly due to the covariation with other traits. Using YLD as an example, we observe a large proportion of direct effect in ΔZ but a slightly higher proportion of indirect over direct effect in S and i. This result suggests that the selection force on YLD receives a higher contribution from selection on other traits more than selection on the trait itself. However, the selection response in YLD receives little to no selection response that can be attributed due to covariation with other traits. In another example with HT, we find positive direct effects in ΔZ, S and i, which contradicts the known selection on dwarfing genes like *Rht1*, *Rht2* and *Rht24* (Pearce et al. 2011, Würschum et al.

2017). Given the uncertainties in the multivariate selection parameters, it is best to treat these estimates with caution as we explore the cause of issues later using simulation.

To test the consistency in multivariate selection parameters across genotyping platforms, we applied the same approach to the TG panel that was genotyped using the Diversity Array Technology (DArT) (Bentley et al. 2014). First, the comparison of G's and g's between GBS and DArT showed highly significant similarity in both Random Skewers (Cheverud and Marroig 2007) and Mantel tests (Mantel 1976) (Table S10). For consistency, we matched the phenotypic data from Bentley et al. (2014) to the DArT marker data and phenotypic data from Ladejobi et al. (2019) to the GBS marker data. The resulting $\Delta\mathrm{Z}$ are the same between the two except for YLD, which has only a small difference. Given that the G and $\Delta\mathrm{Z}$ are highly similar, we expected the β_{sel} to be similar. In contrast, the β_{sel} shared only 4 traits in the same directions and are separated by an angle of 104.6°. As a result, the estimates of S and i, as well as direct and indirect effects, are vastly different (Table S8).

Multivariate selection parameters in a simulated example

To better understand the issues with multivariate selection parameters, we used a simple simulation with a single generation of selection involving three genetically correlated traits. The details of the simulation can be found in the Materials and Methods section. First, we compared G and E between the version estimated from mixed model and the version derived from true simulated values. E is the residual variance-covariance matrix where $\mathrm{P} = \mathrm{G} + \mathrm{E}$. While the means of estimated variances were close to true simulated variance of 1 (Figure S11), the precisions of the estimates were poor (Table S9). Next, we simulated three different types of selection: (1) Sel1: select on individuals with top 10% in trait 1, (2) Sel2: select on individuals with top 10% in trait 1 or 2, (3) Sel3: select on individuals with top 10% in trait 1, 2 or 3.

For each type of selection, we compared the estimated or realized values of $\Delta\mathrm{Z}$, β_{sel}, S and i to the true or expected values. First, we calculated the true values of $\Delta\mathrm{Z}$, β_{sel} and i using the true values for S, G and P. Next, we obtained the realized values of β_{sel}, S and i using the realized $\Delta\mathrm{Z}$ and true G and P. We also obtained the estimated values of β_{sel}, S and i using the realized $\Delta\mathrm{Z}$ and estimated G and P. Across Sel1, Sel2 and Sel3, we found that the correlations were higher between realized and true values than between estimated and true values (Table S9, Figure S12-S17). This result further highlights the issues due to poor estimates of G and P. However, the correlations between the realized and expected values are far from perfect, which suggest that the discrepancies between observed and expected $\Delta\mathrm{Z}$ are sufficient to distort the realized values of β_{sel}, S and i. Lastly, we observed that the correlations were generally higher in Sel1, followed by Sel2 and Sel3 (Table S9). This may suggest that the multivariate selection parameters get poorer as more traits are directly selected.

Supplementary Tables

Table S1. Parametric control estimates and percent significant markers.

Maximum likelihood estimates (MLE) of the mean (δ) and standard deviation (σ) for model correction using Parametric Control (PC) at various allele frequency change thresholds (t = 0.05 to 0.50). The values are shown as the mean and range (in parentheses) across n-simulations with converged MLE (conv.). The first row with t = 0.00 is from the uncorrected model. S: selected, U: unselected population.

	δ		σ		Significant markers (%)		
t	S	U	S	U	S	U	conv.
0.00	NA	NA	NA	NA	1.942 (0.858, 3.535)	0.089 (0, 0.391)	NA
0.05	0.0002 (-0.0143, 0.0162)	0.0006 (-0.0157, 0.0143)	0.5994 (0.5470, 0.6529)	0.5814 (0.5498, 0.6079)	11.152 (8.013, 14.393)	6.391 (4.042, 9.988)	100
0.06	-0.0004 (-0.0192, 0.0178)	0.0007 (-0.0199, 0.0194)	0.6875 (0.6502, 0.7467)	0.6656 (0.6292, 0.7020)	7.284 (4.418, 9.943)	3.212 (1.795, 5.012)	100
0.07	-0.0001 (-0.0219, 0.0220)	0.0012 (-0.0209, 0.0204)	0.7633 (0.7182, 0.8116)	0.7398 (0.6955, 0.7812)	5.093 (2.849, 7.578)	1.613 (0.707, 2.706)	100
0.08	0 (-0.0205, 0.0229)	0.0007 (-0.0210, 0.0188)	0.8341 (0.7941, 0.8825)	0.8086 (0.7710, 0.8498)	3.723 (1.945, 5.833)	0.819 (0.281, 1.385)	100
0.09	-0.0008 (-0.0188, 0.0231)	0.0003 (-0.0215, 0.0196)	0.8990 (0.8556, 0.9518)	0.8710 (0.8319, 0.9096)	2.857 (1.476, 4.591)	0.414 (0.060, 1.039)	98
0.10	-0.0007 (-0.0248, 0.0220)	0.0000 (-0.0202, 0.0223)	0.9578 (0.9105, 1.0068)	0.9270 (0.8917, 0.9713)	2.297 (1.117, 3.798)	0.218 (0.011, 0.557)	96
0.11	0.0002 (-0.0229, 0.0217)	0.0001 (-0.0217, 0.0222)	1.0128 (0.9622, 1.0589)	0.9791 (0.9411, 1.0207)	1.867 (0.961, 3.097)	0.109 (0, 0.440)	92
0.12	-0.0003 (-0.0218, 0.0196)	-0.0003 (-0.0224, 0.0205)	1.0645 (1.0096, 1.1169)	1.0270 (0.9899, 1.0776)	1.554 (0.750, 2.692)	0.058 (0, 0.272)	92
0.13	0.0001 (-0.0238, 0.0186)	0.0001 (-0.0189, 0.0207)	1.1127 (1.0504, 1.1702)	1.0724 (1.0306, 1.1236)	1.313 (0.618, 2.506)	0.029 (0, 0.239)	93
0.14	-0.0002 (-0.0200, 0.0217)	0.0007 (-0.0231, 0.0213)	1.1578 (1.0935, 1.2130)	1.1140 (1.0709, 1.1787)	1.142 (0.476, 2.177)	0.017 (0, 0.147)	94
0.15	0.0002 (-0.0212, 0.0213)	0.0007 (-0.0223, 0.0228)	1.2002 (1.1327, 1.2609)	1.1519 (1.1042, 1.2186)	0.994 (0.391, 2.007)	0.012 (0, 0.092)	93
0.16	0.0003 (-0.0237, 0.0224)	0.0007 (-0.0212, 0.0208)	1.2393 (1.1718, 1.3012)	1.1888 (1.1337, 1.2567)	0.882 (0.332, 1.848)	0.007 (0, 0.065)	95
0.17	0.0001 (-0.0248, 0.0218)	0.0011 (-0.0196, 0.0221)	1.2780 (1.2161, 1.3454)	1.2228 (1.1612, 1.2916)	0.791 (0.230, 1.607)	0.003 (0, 0.054)	95
0.18	0.0002 (-0.0233, 0.0198)	0.0007 (-0.0231, 0.0233)	1.3133 (1.2562, 1.3828)	1.2536 (1.1898, 1.3298)	0.709 (0.197, 1.431)	0.002 (0, 0.033)	96
0.19	0.0002 (-0.0216, 0.0200)	0.0012 (-0.0200, 0.0237)	1.3457 (1.2894, 1.4203)	1.2818 (1.2121, 1.3634)	0.634 (0.170, 1.146)	0.001 (0, 0.027)	96
0.20	-0.0004 (-0.0231, 0.0204)	0.0008 (-0.0185, 0.0256)	1.3758 (1.3185, 1.4528)	1.3074 (1.2363, 1.3972)	0.569 (0.142, 1.066)	0 (0, 0.016)	96
0.21	-0.0008 (-0.0245, 0.0223)	0.0007 (-0.0215, 0.0252)	1.4049 (1.3440, 1.4807)	1.3303 (1.2555, 1.4247)	0.521 (0.137, 1.050)	0 (0, 0.016)	97
0.22	-0.0010 (-0.0261, 0.0215)	0.0009 (-0.0226, 0.0263)	1.4325 (1.3667, 1.5127)	1.3524 (1.2679, 1.4496)	0.470 (0.109, 0.957)	0 (0, 0.005)	97
0.23	-0.0007 (-0.0250, 0.0257)	0.0011 (-0.0258, 0.0263)	1.4576 (1.3884, 1.5392)	1.3712 (1.2799, 1.4770)	0.430 (0.092, 0.886)	0 (0, 0.005)	98
0.24	-0.0007 (-0.0252, 0.0251)	0.0009 (-0.0275, 0.0269)	1.4817 (1.4103, 1.5636)	1.3882 (1.2889, 1.4984)	0.392 (0.066, 0.760)	0 (0, 0)	98

Table S1 continues to the next page.

Table S1 continues from the previous page.

t	δ S	δ U	σ S	σ U	Significant markers (%) S	Significant markers (%) U	conv.
0.25	-0.0007<br>(-0.0260, 0.0268)	0.0008<br>(-0.0269, 0.0256)	1.5045<br>(1.4262, 1.5918)	1.4028<br>(1.2990, 1.5136)	0.357<br>(0.044, 0.735)	0<br>(0, 0)	98
0.26	-0.0013<br>(-0.0270, 0.0257)	0.0007<br>(-0.0303, 0.0241)	1.5251<br>(1.4464, 1.6161)	1.4162<br>(1.3064, 1.5332)	0.330<br>(0.027, 0.697)	0<br>(0, 0)	97
0.27	-0.0013<br>(-0.0288, 0.0243)	0.0005<br>(-0.0293, 0.0242)	1.5450<br>(1.4696, 1.6380)	1.4280<br>(1.3107, 1.5512)	0.303<br>(0.005, 0.659)	0<br>(0, 0)	98
0.28	-0.0011<br>(-0.0294, 0.0269)	0.0004<br>(-0.0296, 0.0252)	1.5625<br>(1.4897, 1.6603)	1.4386<br>(1.3163, 1.5714)	0.284<br>(0.005, 0.648)	0<br>(0, 0)	98
0.29	-0.0014<br>(-0.0295, 0.0253)	0.0004<br>(-0.0290, 0.0228)	1.5790<br>(1.5032, 1.6778)	1.4477<br>(1.3199, 1.5832)	0.265<br>(0.005, 0.611)	0<br>(0, 0)	98
0.30	-0.0013<br>(-0.0293, 0.0248)	0.0003<br>(-0.0287, 0.0239)	1.5943<br>(1.5109, 1.6939)	1.4549<br>(1.3229, 1.5957)	0.251<br>(0.005, 0.606)	0<br>(0, 0)	98
0.31	-0.0012<br>(-0.0310, 0.0267)	0.0004<br>(-0.0267, 0.0237)	1.6088<br>(1.5185, 1.7096)	1.4616<br>(1.3243, 1.6066)	0.235<br>(0.005, 0.578)	0<br>(0, 0)	98
0.32	-0.0013<br>(-0.0308, 0.0237)	0.0001<br>(-0.0271, 0.0239)	1.6224<br>(1.5267, 1.7176)	1.4672<br>(1.3295, 1.6194)	0.221<br>(0, 0.567)	0<br>(0, 0)	98
0.33	-0.0008<br>(-0.0325, 0.0333)	0.0001<br>(-0.0282, 0.0236)	1.6344<br>(1.5421, 1.7337)	1.4720<br>(1.3303, 1.6227)	0.212<br>(0, 0.540)	0<br>(0, 0)	99
0.34	-0.0008<br>(-0.0324, 0.0320)	0.0001<br>(-0.0279, 0.0253)	1.6458<br>(1.5479, 1.7446)	1.4756<br>(1.3327, 1.6260)	0.199<br>(0, 0.535)	0<br>(0, 0)	99
0.35	-0.0010<br>(-0.0322, 0.0303)	0.0001<br>(-0.0279, 0.0249)	1.6567<br>(1.5580, 1.7542)	1.4787<br>(1.3327, 1.6308)	0.191<br>(0, 0.524)	0<br>(0, 0)	99
0.36	-0.0011<br>(-0.0348, 0.0305)	0.0001<br>(-0.0286, 0.0252)	1.6671<br>(1.5693, 1.7683)	1.4810<br>(1.3327, 1.6329)	0.182<br>(0, 0.518)	0<br>(0, 0)	99
0.37	-0.0010<br>(-0.0336, 0.0297)	0.0002<br>(-0.0286, 0.0252)	1.6768<br>(1.5755, 1.7795)	1.4831<br>(1.3327, 1.6366)	0.174<br>(0, 0.513)	0<br>(0, 0)	99
0.38	-0.0010<br>(-0.0338, 0.0296)	0.0002<br>(-0.0291, 0.0257)	1.6857<br>(1.5807, 1.7894)	1.4848<br>(1.3342, 1.6374)	0.166<br>(0, 0.502)	0<br>(0, 0)	99
0.39	-0.0011<br>(-0.0356, 0.0298)	0.0002<br>(-0.0286, 0.0257)	1.6940<br>(1.5849, 1.8016)	1.4860<br>(1.3342, 1.6383)	0.159<br>(0, 0.497)	0<br>(0, 0)	99
0.40	-0.0012<br>(-0.0402, 0.0279)	0.0001<br>(-0.0286, 0.0259)	1.7024<br>(1.5867, 1.8220)	1.4872<br>(1.3342, 1.6402)	0.151<br>(0, 0.497)	0<br>(0, 0)	99
0.41	-0.0012<br>(-0.0409, 0.0275)	0.0000<br>(-0.0286, 0.0259)	1.7098<br>(1.5885, 1.8439)	1.4879<br>(1.3342, 1.6409)	0.146<br>(0, 0.480)	0<br>(0, 0)	99
0.42	-0.0010<br>(-0.0392, 0.0265)	0.0001<br>(-0.0286, 0.0259)	1.7174<br>(1.5984, 1.8578)	1.4887<br>(1.3342, 1.6420)	0.14<br>(0, 0.475)	0<br>(0, 0)	99
0.43	-0.0009<br>(-0.0386, 0.0270)	0.0001<br>(-0.0286, 0.0259)	1.7242<br>(1.6004, 1.8697)	1.4891<br>(1.3342, 1.6420)	0.135<br>(0, 0.469)	0<br>(0, 0)	99
0.44	-0.0010<br>(-0.0371, 0.0260)	0.0000<br>(-0.0286, 0.0259)	1.7304<br>(1.6026, 1.8788)	1.4894<br>(1.3342, 1.6425)	0.131<br>(0, 0.453)	0<br>(0, 0)	99
0.45	-0.0010<br>(-0.0371, 0.0292)	0.0000<br>(-0.0286, 0.0259)	1.7363<br>(1.6113, 1.8939)	1.4896<br>(1.3342, 1.6425)	0.127<br>(0, 0.447)	0<br>(0, 0)	99
0.46	-0.0009<br>(-0.0358, 0.0301)	0.0000<br>(-0.0286, 0.0259)	1.7420<br>(1.6128, 1.9033)	1.4898<br>(1.3342, 1.6425)	0.123<br>(0, 0.442)	0<br>(0, 0)	99
0.47	-0.0008<br>(-0.0374, 0.0294)	0.0000<br>(-0.0286, 0.0259)	1.7480<br>(1.6199, 1.9128)	1.4900<br>(1.3342, 1.6425)	0.120<br>(0, 0.440)	0<br>(0, 0)	99
0.48	-0.0009<br>(-0.0384, 0.0288)	0.0001<br>(-0.0286, 0.0259)	1.7535<br>(1.6219, 1.9196)	1.4902<br>(1.3342, 1.6425)	0.117<br>(0, 0.440)	0<br>(0, 0)	99
0.49	-0.0011<br>(-0.0386, 0.0304)	0.0001<br>(-0.0286, 0.0259)	1.7590<br>(1.6279, 1.9325)	1.4903<br>(1.3342, 1.6425)	0.114<br>(0, 0.440)	0<br>(0, 0)	99
0.50	-0.0011<br>(-0.0382, 0.0307)	0.0000<br>(-0.0286, 0.0259)	1.7647<br>(1.6309, 1.9376)	1.4904<br>(1.3342, 1.6425)	0.110<br>(0, 0.440)	0<br>(0, 0)	99

Table S2. Genomic positions of 50 GWAS QTLs in the TG panel.

The IWGSC RefSeq v1.0 physical positions of the peaks and boundaries (Ladejobi et al. 2019) are clustered into groups according to their positions. The traits associated with each group are listed along with the percent variance explained (PVE). The overlapping QSLs from RALLY in TG and WAGTAIL panels are also shown, which correspond to the QSL numbers in the first columns of Table 1 and S4.

QTL	Chr	Position (bp)			Trait (PVE)	Overlapping QSL/QTL	
		Start	End	Peak		TG	WAGTAIL
1	1A	-	-	42,151,448	PROT (2.9)	1	0
2	1A	532,433,018	532,529,494	532,529,494	AWNS (4.3)	0	0
3	1B	-	-	4,341,220	FT (4.1)	0	0
4	1B	40,429,328	40,806,011	40,806,011	FT (3.7)	0	0
5	1B	628,112,579	636,796,017	628,112,579	AWNS (9.6)	0	0
6	1D	-	-	343,103,677	AWNS (3.2)	0	0
7	2A	-	-	3,634,548	LODG (3.9)	4	5
8	2A	-	-	748,997,740	YLD (4.0)	0	0
9	2B	-	-	1,467,644	LODG (4.8)	0	0
10	2B	688,433,192	688,433,222	688,433,192	FT (4.0)	6	9
11	2B	790,828,845	800,780,364	794,977,501	AWNS (3.7)	0	0
12	2D	-	-	7,738,369	AWNS (2.9)	0	0
13	2D	-	-	8,787,004	LODG (5.0)	0	0
14	2D	-	-	31,468,893	FT (9.6), HT (3.5), MAT (6.1)	0	0
15	2D	-	-	42,097,013	FT (9.3), HT (3.9), MAT (7.2)	0	0
16	2D	572,734,652	608,800,104	572,918,169	LODG (5.0)	0	0
17	3A	-	-	161,444,257	AWNS (3.2)	0	0
18	3A	-	-	463,564,378	LODG (3.5)	0	0
19	3A	-	-	495,524,635	HT (2.9)	7	13
20	3B	-	-	5,601,689	PROT (2.7)	0	0
21	4A	222,595,975	298,655,753	298,655,753	LODG (3.7)	0	14
22	4A	639,058,378	729,741,651	639,058,378	PROT (3.1)	10	0
22	4A	719,113,347	729,872,889	719,113,347	AWNS (3.5)	0	0
23	4A	743,082,595	744,433,737	743,226,828	HT (3.4)	0	0
24	4B	21,378,087	21,379,808	21,378,087	HT (4.2)	0	0
25	4B	-	-	55,242,733	WK (3.7)	0	0
26	4B	-	-	520,086,297	LODG (4.1)	11	0
27	4B	-	-	591,282,163	AWNS (3.3)	11	0
28	5A	59,536,488	67,301,668	63,500,759	WK (4.6)	12	16
29	5A	665,971,659	685,792,099	679,803,244	HT (3.1)	0	0
29	5A	680,532,103	709,755,448	708,820,599	FT (3.9), AWNS (28.9)	0	0
30	5B	-	-	107,367,824	PROT (3.8)	0	0
31	5B	580,426,360	588,757,384	588,757,384	AWNS (5.3)	0	0
32	6A	3,758,004	21,719,436	16,462,141	AWNS (10.8)	15	0
33	6A	85,580,220	91,681,786	88,260,960	YLD (3.9)	16	18
34	6A	101,430,018	101,851,274	101,430,018	HT (4.5), LODG (4.4)	16	18
34	6A	85,263,886	115,457,176	112,688,966	PROT (5.5)	16	18
35	6A	-	-	151,492,080	PROT (2.8)	16	18
36	6A	373,461,190	452,372,111	422,756,419	HT (5.0)	16	18
36	6A	416,675,591	448,706,137	448,010,707	PROT (3.8)	16	18

36	6A	373,461,190	450,106,742	450,106,742	LODG (6.1)	16	18
37	6A	453,334,056	461,434,500	454,675,620	HT (4.5)	16	18
37	6A	454,675,620	465,641,292	465,641,261	LODG (4.7)	16	18
38	6A	614,089,848	615,386,089	614,663,042	FT (3.6)	17	0
39	6B	-	-	106,381,266	PROT (3.9)	0	0
40	6D	-	-	2,449,348	LODG (3.7)	0	0
41	6D	-	-	5,155,743	AWNS (6.3)	0	0
42	6D	461,923,517	462,303,873	461,923,517	FT (4.5)	0	0
43	7A	14,418,771	15,750,691	15,031,731	HT (3.5)	0	19
43	7A	14,418,771	22,872,792	15,639,304	FT (6.6)	0	19
44	7A	-	-	62,185,902	PROT (2.8)	0	0
45	7A	-	-	278,811,242	LODG (3.4)	0	0
46	7A	-	-	695,324,003	YLD (3.4)	0	0
47	7B	-	-	11,703,887	HT (3.0)	0	0
48	7B	45,162,103	65,376,987	45,162,103	LODG (4.3)	21	0
48	7B	50,826,708	63,170,244	63,134,685	YLD (3.7), PROT (3.7)	21	0
49	7B	678,635,628	702,820,571	678,635,628	LODG (3.7)	22	0
49	7B	-	-	702,820,571	HT (2.5)	22	0
50	7D	-	-	16,704,573	FT (4.9)	0	0

Table S3. Co-localization between RALLY QSLs and others.

QTLs identified from three recent studies in wheat are compared against the RALLY QSLs. All physical positions are listed according to the IWGSC RefSeq v1.0, except for the meta QTLs by Yang et al. (2021) which have not been explicitly stated.

QSL	Position (chromosome + interval in Mb)	QTL co-localization (chromosome + interval in Mb)		
		MAGIC diverse GWAS (Scott et al. 2021)	Meta QTL (Yang et al. 2021)	Introgression (Cheng et al. 2019)
1	1A (36.8 - 42.2)	-	-	-
2	1A (105.9 - 395.5)	1A (389.0 - 468.4)	-	1A (210.3 - 233.7)
3	1B (52.8 - 573.0)	1B (95.6 - 566.7)	1B-4 (542.9 - 563.1)	1B (560.0 - 560.1)
4	2A (0.5 - 36.1)	Yr17	2A-2 (4.0 - 10.0), 2A-3 (28.0 - 30.5), 2A-4 (33.3 - 34.5)	2A (0.0 - 11.0)
5	2A (56.2 - 115.4)	-	2A-1 (59.4 - 71.6)	-
6	2B (10.9 - 785.6)	Ppd-B1, Yr7/Yr5/YrSP	2B-3 (50.7 - 80.1), 2B-4 (104.1 - 730.2)	2B (89.6 - 748.7)
7	3A (488.3 - 574.6)	-	3A-3 (471.4 - 533.6)	3A (508.3 - 509.4)
8	3B (19.1 - 31.4)	-	3B-3 (12.3 - 26.8)	-
9	3B (813.3 - 830.0)	-	-	3B (822.4 - 829.7)
10	4A (507.7 - 695.9)	4A (599.1 - 610.1)	4A-2 (606.7 - 611.1), 4A-3 (591.0 - 597.1), 4A-4 (618.3 - 621.7), 4A-5 (616.6 - 626.8), 4A-6 (654.5 - 668.8)	4A (372.4 - 679.2)
11	4B (507.2 - 593.8)	-	-	-
12	5A (31.1 - 449.8)	5A (13.7 - 34.2)	-	-
13	5B (681.3 - 703.9)	-	5B-6 (679.4 - 692.6)	-
14	5D (43.4 - 233.7)	-	5D-3 (44.6 - 54.7), 5D-5 (48.7 - 80.3)	5D (188.3 - 188.8)
15	6A (0.7 - 5.1)	-	6A-1 (0.3 - 6.7)	-
16	6A (61.8 - 545.4)	TaGW2, Rht24	6A-3 (58.2 - 85.8), 6A-4 (518.8 - 538.4)	6A (281.5 - 282.0)
17	6A (596.6 - 617.3)	6A (609.3 - 618.3)	6A-7 (595.0 - 602.9), 6A-8 (606.6 - 615.1)	6A (596.2 - 598.6)
18	7A (610.2 - 612.6)	-	7A-5 (108.8 - 616.6)	-
19	7A (669.8 - 695.0)	WAPO-A1	7A-6 (675.3 - 688.7), 7A-7 (694.4 - 703.6)	7A (669.1 - 698.6)
20	7B (3.4 - 4.8)	-	7B-1 (1.0 - 6.2)	-
21	7B (40.3 - 58.9)	-	-	-
22	7B (698.2 - 707.9)	-	7B-6 (697.5 - 707.7)	-

Table S4. Genomic positions of 19 RALLY QSLs in the WAGTAIL panel.

The IWGSC RefSeq v1.0 physical positions of the peaks and LD boundaries are shown, along with the P-values associated with the peaks and overlapping QSLs/QTLs from RALLY in TG panel and GWAS in TG panel (Ladejobi et al. 2019). The numbers in the overlapping QSLs/QTLs columns correspond to the QSL and QTL numbers in the first columns of Table 1 and S4.

QSL	Chr	Position (bp)			$-\log_{10}P$	Overlapping QSL/QTL	
		Start	End	Peak		TG	GWAS
1	1A	11,950,553	1,336,994	14,253,976	7.173	0	0
2	1A	462,001,757	38,729,508	490,087,895	11.148	1,2	0
3	1B	303,680,331	303,680,331	571,707,456	5.665	3	0
4	1D	56,751,172	34,621,416	56,753,155	8.717	0	0
5	2A	24,278,890	2,646,023	32,139,650	7.634	4	7
6	2A	59,558,265	58,394,953	77,163,127	5.429	5	0
7	2A	639,988,472	605,184,949	676,977,112	9.025	0	0
8	2A	762,503,117	762,292,207	771,236,862	6.997	0	0
9	2B	146,634,773	44,922,004	773,349,577	9.882	6	10
10	2B	784,551,365	784,551,365	790,751,851	5.198	6	0
11	2D	15,967,398	14,778,701	18,234,286	5.506	0	0
12	3A	106,435,192	86,369,509	107,738,831	6.884	0	0
13	3A	497,649,046	495,073,247	573,985,537	7.685	7	19
14	4A	590,123,186	68,470,282	599,846,393	8.838	10	21
15	4B	645,298,612	634,340,134	652,775,661	5.135	0	0
16	5A	118,667,316	32,880,888	450,555,721	7.028	12	28
17	5B	678,337,596	663,522,311	679,687,636	6.331	0	0
18	6A	409,092,614	61,813,444	582,253,761	9.596	16	33,34,35,36,37
19	7A	35,770,406	20,294,545	47,283,220	5.260	0	43
20	1A	11,950,553	1,336,994	14,253,976	7.173	0	0
21	1A	462,001,757	38,729,508	490,087,895	11.148	1,2	0
22	1B	303,680,331	303,680,331	571,707,456	5.665	3	0

Table S5. Likelihood ratio tests for local heritabilities

Mixed linear models with and without the QSLs were compared using a likelihood ratio test (LRT) similar to the approach of Santantonio et al. (2019). The test statistic is $\chi^2_{df=1} = 2 \cdot (LL_1 - LL_0)$ where LL_1 is the log-likelihood of the model with QSL and LL_0 is the log-likelihood of the model without QSL. P-values are shown here.

QSL	FT	LODG	YLD	HT	PROT	WK	AWNS	SPWT	TGW	EM2	TILL	MAT
1	1.000	0.998	0.936	0.087	0.159	0.171	0.999	1.000	1.000	0.324	0.999	1.000
2	1.000	0.450	0.140	0.047	0.552	0.999	1.000	0.824	0.235	0.272	1.000	1.000
3	1.000	0.994	0.615	0.993	0.482	0.998	0.867	0.868	1.000	0.741	0.763	0.462
4	1.000	0.995	0.997	0.994	0.998	0.998	0.570	0.999	1.000	1.000	0.031	1.000
5	1.000	0.142	1.000	0.298	0.998	1.000	0.995	0.886	1.000	1.000	0.999	1.000
6	1.000	0.976	0.181	1.000	1.000	0.546	0.999	0.998	0.367	1.000	0.686	0.063
7	1.000	0.994	0.932	0.992	0.673	1.000	1.000	0.999	0.408	0.996	0.192	1.000
8	0.566	0.994	1.000	0.570	0.998	0.998	0.996	0.193	0.997	0.993	1.000	1.000
9	1.000	0.855	0.763	0.996	0.360	0.998	1.000	1.000	0.996	0.994	0.576	1.000
10	0.519	0.448	1.000	0.992	0.998	0.663	0.586	0.276	0.114	0.728	0.170	0.063
11	1.000	0.062	0.049	0.892	0.762	0.522	0.390	0.255	0.241	0.994	0.999	1.000
12	1.000	0.779	0.497	0.992	0.488	0.531	0.183	0.745	0.996	0.607	1.000	0.893
13	1.000	0.994	0.756	0.840	1.000	0.208	0.997	1.000	0.306	0.993	1.000	1.000
14	0.295	0.996	0.152	0.992	0.038	0.524	1.000	0.933	0.995	0.996	0.999	0.380
15	0.469	0.996	1.000	0.992	1.000	0.406	0.368	0.909	0.332	0.418	1.000	1.000
16	1.000	0.097	2.97E-03	1.64E-06	1.73E-04	0.999	0.997	0.085	0.184	0.361	0.999	0.589
17	0.064	0.994	0.515	0.992	0.744	0.047	1.000	0.999	0.820	0.993	0.279	0.218
18	1.000	0.994	0.333	0.997	0.999	1.000	0.995	1.000	0.831	0.996	0.853	1.000
19	1.000	0.374	0.755	0.993	1.000	0.998	0.270	0.999	0.016	0.704	1.000	1.000
20	1.000	0.994	0.225	0.101	0.434	0.786	1.000	1.000	0.994	1.000	1.000	1.000
21	1.000	8.45E-03	1.000	0.397	1.000	0.998	1.000	0.378	0.374	0.993	1.000	1.000
22	0.454	0.997	0.845	0.206	1.000	1.000	0.991	1.000	0.109	0.993	0.110	1.000
All	0.020	1.87E-03	8.22E-05	1.65E-07	1.67E-05	0.041	0.092	0.041	4.51E-03	0.120	1.19E-03	6.67E-03

Table S6. Counts of pairwise RR effects for alleles that are increasing over time.

For each pair of traits, the number of markers with positive and negative RR effects are counted for the increasing alleles.

trait pair	+/+	+/-	-/+	-/-	trait pair	+/+	+/-	-/+	-/-
FT/LODG	7,750	11,547	6,580	12,959	HT/SPWT	8,336	7,027	10,862	12,611
FT/YLD	12,996	6,301	13,251	6,288	HT/TGW	6,984	8,379	10,259	13,214
FT/HT	10,552	8,745	4,811	14,728	HT/EM2	7,491	7,872	12,107	11,366
FT/PROT	8,153	11,144	6,851	12,688	HT/TILL	7,068	8,295	12,408	11,065
FT/WK	6,738	12,559	11,821	7,718	HT/MAT	9,382	5,981	10,916	12,557
FT/AWNS	7,926	11,371	11,880	7,659	PROT/WK	6,824	8,180	11,735	12,097
FT/SPWT	9,474	9,823	9,724	9,815	PROT/AWNS	7,855	7,149	11,951	11,881
FT/TGW	6,972	12,325	10,271	9,268	PROT/SPWT	8,430	6,574	10,768	13,064
FT/EM2	9,472	9,825	10,126	9,413	PROT/TGW	6,380	8,624	10,863	12,969
FT/TILL	8,408	10,889	11,068	8,471	PROT/EM2	6,887	8,117	12,711	11,121
FT/MAT	15,617	3,680	4,681	14,858	PROT/TILL	7,520	7,484	11,956	11,876
LODG/YLD	8,729	5,601	17,518	6,988	PROT/MAT	8,019	6,985	12,279	11,553
LODG/HT	8,999	5,331	6,364	18,142	WK/AWNS	9,824	8,735	9,982	10,295
LODG/PROT	5,832	8,498	9,172	15,334	WK/SPWT	8,788	9,771	10,410	9,867
LODG/WK	6,501	7,829	12,058	12,448	WK/TGW	8,666	9,893	8,577	11,700
LODG/AWNS	7,201	7,129	12,605	11,901	WK/EM2	9,439	9,120	10,159	10,118
LODG/SPWT	7,255	7,075	11,943	12,563	WK/TILL	9,156	9,403	10,320	9,957
LODG/TGW	6,624	7,706	10,619	13,887	WK/MAT	8,542	10,017	11,756	8,521
LODG/EM2	6,883	7,447	12,715	11,791	AWNS/SPWT	10,706	9,100	8,492	10,538
LODG/TILL	7,411	6,919	12,065	12,441	AWNS/TGW	9,840	9,966	7,403	11,627
LODG/MAT	7,390	6,940	12,908	11,598	AWNS/EM2	10,468	9,338	9,130	9,900
YLD/HT	9,905	16,342	5,458	7,131	AWNS/TILL	11,302	8,504	8,174	10,856
YLD/PROT	6,336	19,911	8,668	3,921	AWNS/MAT	8,454	11,352	11,844	7,186
YLD/WK	11,905	14,342	6,654	5,935	SPWT/TGW	8,588	10,610	8,655	10,983
YLD/AWNS	13,387	12,860	6,419	6,170	SPWT/EM2	10,571	8,627	9,027	10,611
YLD/SPWT	13,237	13,010	5,961	6,628	SPWT/TILL	9,178	10,020	10,298	9,340
YLD/TGW	12,504	13,743	4,739	7,850	SPWT/MAT	9,157	10,041	11,141	8,497
YLD/EM2	13,891	12,356	5,707	6,882	TGW/EM2	6,917	10,326	12,681	8,912
YLD/TILL	12,962	13,285	6,514	6,075	TGW/TILL	9,080	8,163	10,396	11,197
YLD/MAT	14,055	12,192	6,243	6,346	TGW/MAT	7,533	9,710	12,765	8,828
HT/PROT	7,220	8,143	7,784	15,689	EM2/TILL	9,843	9,755	9,633	9,605
HT/WK	5,846	9,517	12,713	10,760	EM2/MAT	9,964	9,634	10,334	8,904
HT/AWNS	7,036	8,327	12,770	10,703	TILL/MAT	9,852	9,624	10,446	8,914

Table S7. Counts of pairwise LASSO effects for alleles that are increasing over time.

For each pair of traits, the number of markers with positive and negative LASSO effects are counted for the increasing alleles. Significance for each pair of traits (contingency table) is determined from a $\chi^2_{df=1}$ distribution.

trait pair	+/+	+/-	-/+	-/-	$-\log_{10}p$	trait pair	+/+	+/-	-/+	-/-	$-\log_{10}p$
FT/LODG	90	223	41	163	1.456	HT/SPWT	71	63	149	234	2.206
FT/YLD	270	43	145	59	4.435	HT/TGW	68	66	148	235	1.719
FT/HT	112	201	22	182	9.352	HT/EM2	56	78	214	169	2.170
FT/PROT	77	236	59	145	0.491	HT/TILL	74	60	212	171	0.000
FT/WK	135	178	135	69	6.326	HT/MAT	102	32	223	160	3.474
FT/AWNS	139	174	140	64	6.959	PROT/WK	57	79	213	168	2.165
FT/SPWT	117	196	103	101	2.367	PROT/AWNS	67	69	212	169	0.624
FT/TGW	120	193	96	108	1.215	PROT/SPWT	82	54	138	243	5.742
FT/EM2	151	162	119	85	1.506	PROT/TGW	64	72	152	229	0.754
FT/TILL	170	143	116	88	0.200	PROT/EM2	64	72	206	175	0.717
FT/MAT	268	45	57	147	38.908	PROT/TILL	77	59	209	172	0.097
LODG/YLD	101	30	314	72	0.452	PROT/MAT	81	55	244	137	0.388
LODG/HT	75	56	59	327	20.083	WK/AWNS	145	125	134	113	0.013
LODG/PROT	40	91	96	290	0.607	WK/SPWT	96	174	124	123	2.977
LODG/WK	64	67	206	180	0.368	WK/TGW	124	146	92	155	1.250
LODG/AWNS	55	76	224	162	2.688	WK/EM2	148	122	122	125	0.598
LODG/SPWT	55	76	165	221	0.018	WK/TILL	166	104	120	127	2.370
LODG/TGW	67	64	149	237	1.801	WK/MAT	161	109	164	83	0.874
LODG/EM2	59	72	211	175	1.148	AWNS/SPWT	135	144	85	153	2.313
LODG/TILL	77	54	209	177	0.385	AWNS/TGW	119	160	97	141	0.137
LODG/MAT	83	48	242	144	0.011	AWNS/EM2	160	119	110	128	1.829
YLD/HT	106	309	28	74	0.103	AWNS/TILL	169	110	117	121	1.922
YLD/PROT	74	341	62	40	17.486	AWNS/MAT	145	134	180	58	7.317
YLD/WK	213	202	57	45	0.324	SPWT/TGW	85	135	131	166	0.607
YLD/AWNS	223	192	56	46	0.036	SPWT/EM2	148	72	122	175	8.195
YLD/SPWT	176	239	44	58	0.007	SPWT/TILL	105	115	181	116	2.427
YLD/TGW	170	245	46	56	0.286	SPWT/MAT	119	101	206	91	3.269
YLD/EM2	228	187	42	60	1.765	TGW/EM2	85	131	185	116	5.962
YLD/TILL	230	185	56	46	0.000	TGW/TILL	122	94	164	137	0.144
YLD/MAT	280	135	45	57	4.687	TGW/MAT	125	91	200	101	1.239
HT/PROT	56	78	80	303	5.408	EM2/TILL	146	124	140	107	0.213
HT/WK	34	100	236	147	11.996	EM2/MAT	149	121	176	71	3.643
HT/AWNS	49	85	230	153	5.361	TILL/MAT	177	109	148	83	0.170

Table S8. Selection gradients, responses, differentials and intensities.

Selection gradients (β_{sel}) were estimated from the multivariate breeder's equation of $\Delta Z = G\beta_{sel}$ using the genetic variance-covariance (G) calculated from the 12 traits. Selection responses (ΔZ) were estimated from regressing the traits over year of release. Selection differentials (S) and intensity (i) were calculated from $S = P\beta_{sel}$ and $i = S/\sqrt{diag(P)}$ where P is the phenotypic variance-covariance matrix. The estimated values were partitioned into direct and indirect components due to the direct contributions from selection on the trait itself or indirect contributions from selection on other correlated traits.

Data	Trait	β_{sel}	ΔZ			S			i		
			Total	Direct	Indirect	Total	Direct	Indirect	Total	Direct	Indirect
DArT	FT	-0.578	-0.017	-1.383	1.366	-0.199	-2.824	2.626	-0.090	-1.277	1.188
	LODG	0.877	-0.033	0.085	-0.118	0.156	0.323	-0.167	0.257	0.532	-0.275
	YLD	0.233	0.365	1.221	-0.856	2.941	3.934	-0.993	0.716	0.957	-0.242
	HT	-0.024	-0.312	-0.249	-0.064	-0.071	-0.624	0.553	-0.014	-0.123	0.109
	PROT	1.176	-0.028	0.133	-0.161	-0.093	0.323	-0.416	-0.178	0.616	-0.794
	WK	-0.563	-0.004	-0.208	0.203	-0.410	-0.530	0.120	-0.422	-0.546	0.124
	AWNS	0.977	0.003	0.025	-0.022	0.001	0.040	-0.039	0.006	0.198	-0.192
	SPWT	-0.210	-0.001	-0.100	0.099	-0.288	-0.622	0.335	-0.167	-0.362	0.194
	TGW	0.114	-0.017	0.077	-0.094	0.402	0.364	0.038	0.225	0.204	0.021
	EM2	0.023	0.101	4.211	-4.110	17.103	18.234	-1.131	0.604	0.644	-0.040
	TILL	-2.433	0.001	-0.020	0.021	-0.118	-0.180	0.062	-0.435	-0.662	0.228
	MAT	2.029	-0.001	0.352	-0.353	0.340	0.874	-0.534	0.518	1.332	-0.814
GBS	FT	-0.155	-0.017	-0.368	0.351	0.721	-0.775	1.496	0.323	-0.347	0.670
	LODG	-1.116	-0.033	-0.090	0.058	-0.335	-0.412	0.076	-0.552	-0.678	0.126
	YLD	0.071	0.352	0.341	0.011	2.761	1.092	1.669	0.702	0.278	0.424
	HT	0.071	-0.312	0.772	-1.084	0.509	1.753	-1.244	0.103	0.354	-0.251
	PROT	-0.934	-0.028	-0.092	0.064	-0.252	-0.238	-0.014	-0.500	-0.472	-0.028
	WK	-0.317	-0.004	-0.094	0.089	-0.472	-0.290	-0.182	-0.493	-0.303	-0.190
	AWNS	-0.591	0.003	-0.014	0.017	-0.031	-0.022	-0.010	-0.164	-0.113	-0.052
	SPWT	0.471	-0.001	0.266	-0.267	1.269	1.344	-0.075	0.752	0.796	-0.044
	TGW	-0.081	-0.017	-0.054	0.037	-0.061	-0.254	0.194	-0.034	-0.143	0.109
	EM2	-0.010	0.101	-2.529	2.630	-3.661	-7.747	4.086	-0.132	-0.279	0.147
	TILL	0.355	0.001	0.003	-0.002	-0.007	0.026	-0.033	-0.026	0.096	-0.122
	MAT	0.777	-0.001	0.125	-0.126	0.323	0.330	-0.006	0.496	0.506	-0.010

Table S9. Correlations of multivariate breeder's equation variables in simulated data.

Correlations of variables were calculated from 100 simulations between true/expected and estimated values, and between true/expected and realized values. Estimated values are derived from mixed model G and P matrices while realized values are derived from true G and P matrices. G: genetic variance-covariance (vcov), E: residual vcov, P: phenotypic vcov, Z: selection response, β: selection gradient, S: selection differential, i: selection intensity.

Variable		Correlation (true vs. estimated values)			Correlation (true vs. realized values)		
		Trait 1	Trait 2	Trait 3	Trait 1	Trait 2	Trait 3
G	Trait 1	-	-	-	-	-	-
	Trait 2	0.417	-	-	-	-	-
	Trait 3	0.326	0.407	-	-	-	-
E	Trait 1	0.484	-	-	-	-	-
	Trait 2	0.618	0.570	-	-	-	-
	Trait 3	0.446	0.518	0.566	-	-	-
Sel1	Z	-	-	-	0.144	0.857	0.721
	Z, direct	0.230	0.346	0.314	0.069	0.611	0.449
	Z, indirect	0.190	0.213	0.315	0.550	0.756	0.843
	β	0.402	0.360	0.331	0.069	0.611	0.449
	S	0.095	0.460	0.308	0.522	0.795	0.605
	S, direct	0.083	0.344	0.317	0.441	0.618	0.451
	S, indirect	0.294	0.367	0.385	0.591	0.837	0.879
	i	0.289	0.476	0.325	0.155	0.791	0.603
	i, direct	0.270	0.352	0.323	0.154	0.615	0.449
	i, indirect	0.294	0.373	0.394	0.593	0.830	0.877
Sel2	Z	-	-	-	0.609	0.692	0.730
	Z, direct	0.268	0.247	0.209	0.310	0.394	0.458
	Z, indirect	0.174	0.263	0.335	0.738	0.735	0.779
	β	0.314	0.223	0.200	0.310	0.394	0.458
	S	0.304	0.182	0.292	0.637	0.614	0.627
	S, direct	0.204	0.026	0.194	0.450	0.434	0.448
	S, indirect	0.375	0.422	0.333	0.759	0.827	0.839
	i	0.358	0.268	0.321	0.554	0.581	0.637
	i, direct	0.257	0.112	0.196	0.360	0.405	0.453
	i, indirect	0.351	0.421	0.334	0.753	0.822	0.831
Sel3	Z	-	-	-	0.546	0.612	0.448
	Z, direct	0.087	0.205	0.041	0.229	0.359	0.181
	Z, indirect	0.122	0.101	0.355	0.731	0.543	0.677
	β	0.121	0.155	0.084	0.229	0.359	0.181
	S	0.119	0.197	0.030	0.521	0.559	0.342
	S, direct	0.052	0.073	-0.015	0.313	0.409	0.209
	S, indirect	0.390	0.287	0.309	0.810	0.694	0.740
	i	0.130	0.253	0.086	0.450	0.514	0.280
	i, direct	0.076	0.107	0.029	0.257	0.378	0.182
	i, indirect	0.380	0.279	0.314	0.798	0.682	0.735

Table S10. Stability of the genetic variance-covariance (G) and correlation (g) matrices across years of release and countries of origin.

The matrices are compared pairwise using Random Skewers (Cheverud and Marroig 2007) and Mantel test (Mantel 1967). For Random Skewers, the results are shown as the correlations between the predicted responses from the two matrices and the p-values from 1,000 permutations. For Mantel test, the results are shown as the correlations between the off-diagonal elements of the two matrices and the p-values from 10,000 permutations. In both tests, a significance indicates similarity between the two matrices.

Matrix 1	Matrix 2	Random Skewers		Mantel test	
		Correlation	P-value	Correlation	P-value
G	G_{DArT}	0.994	0	0.870	1e-4
g	g_{DArT}	0.941	0	0.899	1e-4
G	G_{DE}	0.924	0	0.543	0.003
G	G_{FR}	0.979	0	-0.001	0.920
G	G_{UK}	0.974	0	0.615	0.002
G	G_{y1}	0.984	0	0.788	1e-4
G	G_{y2}	0.875	0	0.195	0.108
G	G_{y3}	0.978	0	0.300	0.038
G_{DE}	G_{FR}	0.887	0	-0.484	0.003
G_{DE}	G_{UK}	0.891	0	-0.013	0.432
G_{FR}	G_{UK}	0.939	0	-0.203	0.111
G_{y1}	G_{y2}	0.856	0	-0.303	0.081
G_{y1}	G_{y3}	0.977	0	0.438	0.010
G_{y2}	G_{y3}	0.886	0	-0.331	0.029
g	g_{DE}	0.625	0.191	0.449	0.002
g	g_{FR}	0.876	0	0.825	1e-4
g	g_{UK}	0.510	0.852	0.280	0.037
g	g_{y1}	0.761	0	0.700	1e-4
g	g_{y2}	0.755	0	0.624	1e-4
g	g_{y3}	0.660	0.049	0.693	1e-4
g_{DE}	g_{FR}	0.456	0.966	0.223	0.105
g_{DE}	g_{UK}	0.271	1	0.018	0.913
g_{FR}	g_{UK}	0.368	1	0.108	0.430
g_{y1}	g_{y2}	0.434	0.982	0.223	0.068
g_{y1}	g_{y3}	0.491	0.912	0.433	1e-4
g_{y2}	g_{y3}	0.473	0.950	0.437	3e-4

Table S11. Genetic correlation and variance-covariance matrix for 12 traits in the TG panel.

Genetic correlations, variances (highlighted in grey) and covariances are shown in the top right triangle, diagonal and bottom left triangle, respectively.

Marker	Trait	FT	LODG	YLD	HT	PROT	WK	AWNS	SPWT	TGW	EM2	TILL	MAT
DArT	FT	2.394	0.226	0.074	0.409	-0.070	-0.510	-0.215	-0.254	-0.476	-0.039	0.059	0.867
	LODG	0.109	0.097	-0.198	0.596	0.128	-0.309	0.262	0.438	-0.085	-0.188	0.628	0.052
	YLD	0.262	-0.141	5.241	-0.314	-0.768	0.184	0.041	-0.420	0.054	0.033	-0.062	-0.009
	HT	2.034	0.597	-2.309	10.328	0.352	-0.491	0.006	0.455	-0.063	-0.112	0.086	0.198
	PROT	-0.036	0.013	-0.591	0.381	0.113	-0.228	0.024	0.643	0.061	-0.181	0.151	-0.053
	WK	-0.479	-0.059	0.256	-0.959	-0.047	0.369	0.056	-0.134	0.121	0.275	0.224	-0.187
	AWNS	-0.053	0.013	0.015	0.003	0.001	0.005	0.026	0.454	0.270	0.058	0.234	-0.403
	SPWT	-0.271	0.094	-0.665	1.011	0.150	-0.056	0.051	0.477	-0.150	0.295	-0.135	-0.499
	TGW	-0.603	-0.022	0.100	-0.165	0.017	0.060	0.036	-0.085	0.669	-0.517	0.329	-0.433
	EM2	-0.826	-0.798	1.013	-4.916	-0.828	2.275	0.127	2.770	-5.755	185.22	-0.210	-0.130
	TILL	0.008	0.018	-0.013	0.025	0.005	0.012	0.003	-0.008	0.024	-0.257	0.008	0.176
	MAT	0.559	0.007	-0.009	0.266	-0.007	-0.047	-0.027	-0.144	-0.148	-0.738	0.007	0.174
GBS	FT	2.367	0.498	-0.077	0.388	0.053	-0.432	-0.284	-0.205	-0.492	-0.065	-0.009	0.808
	LODG	0.218	0.081	-0.205	0.723	0.048	-0.315	0.094	0.235	-0.522	0.072	0.415	0.102
	YLD	-0.260	-0.128	4.830	-0.461	-0.726	0.259	0.144	-0.214	0.190	0.138	0.081	-0.057
	HT	1.964	0.677	-3.333	10.826	0.395	-0.302	-0.116	0.305	-0.235	-0.042	-0.069	-0.033
	PROT	0.026	0.004	-0.500	0.408	0.098	-0.285	-0.079	0.498	-0.096	-0.202	-0.140	-0.047
	WK	-0.361	-0.049	0.309	-0.540	-0.049	0.295	0.020	-0.021	0.130	0.253	-0.111	-0.095
	AWNS	-0.068	0.004	0.049	-0.059	-0.004	0.002	0.024	0.405	0.319	-0.093	0.380	-0.296
	SPWT	-0.237	0.050	-0.353	0.755	0.117	-0.009	0.047	0.565	-0.293	0.310	0.023	-0.510
	TGW	-0.618	-0.122	0.342	-0.631	-0.025	0.058	0.041	-0.180	0.668	-0.529	0.301	-0.579
	EM2	-1.585	0.326	4.813	-2.199	-1.005	2.176	-0.228	3.686	-6.846	250.92	-0.034	-0.019
	TILL	-0.001	0.011	0.017	-0.022	-0.004	-0.006	0.006	0.002	0.024	-0.051	0.009	0.302
	MAT	0.500	0.012	-0.050	-0.044	-0.006	-0.021	-0.019	-0.154	-0.190	-0.118	0.012	0.162

Supplementary Figures

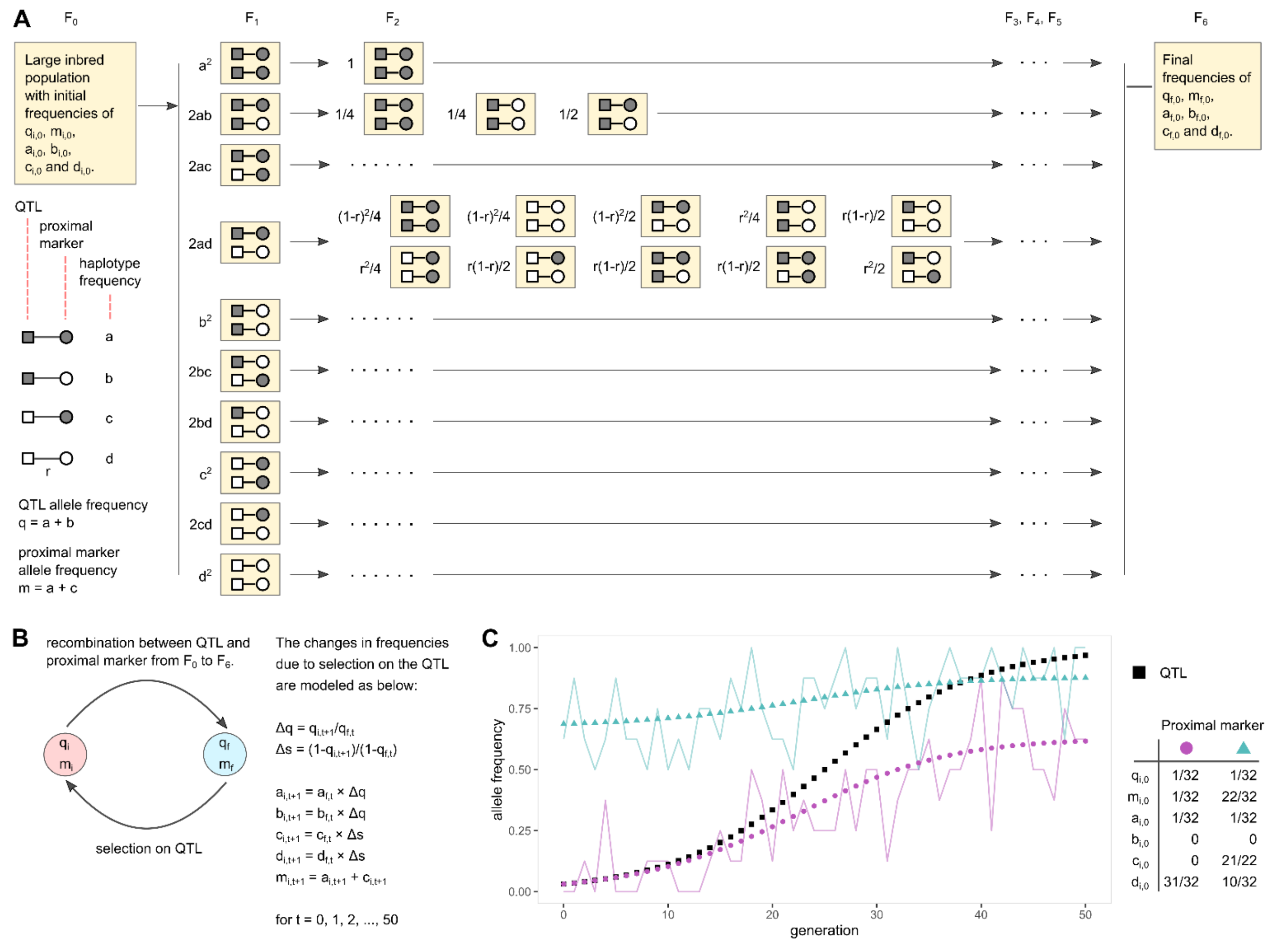

Figure S1. Modelling of hitch-hiking effect due to selection on QTL.

We simulated selection on a QTL and observed the possible hitch-hiking effect in the proximal marker. We used the simulation results to determine the extent of RALLY detection limit in marker linked to QTLs under selection. [A] Given the initial frequencies of the QTL allele (q_i), proximal marker allele (m_i) and QTL-marker haplotypes (a_i, b_i, c_i and d_i) in the parents, we determined the expected frequencies q_f, m_f, a_f, b_f, c_f and d_f in the F_6 individuals. We tested distances of 1, 2, …, 10 cM between the QTL and proximal marker. [B] By modeling selection directly on the QTL as an increase in QTL allele frequency, we computed the correlated changes in proximal marker allele and QTL-marker haplotypes frequencies. [C] An example of a QTL with an initial frequency of 1/32 rising to 31/32 over 50 generations of selection under a logistic model. Two proximal markers that are 1 cM away from the QTL are considered. Initially, the proximal marker allele shown as purple circle is completely linked to the QTL allele while the proximal marker allele shown as blue triangle is weakly linked to the QTL allele. The line plots show the proximal marker allele frequencies from random sampling of 8 individuals per generation. Under the same sample size, the first marker is more likely to be significant under a logistic regression model than the second marker which is largely invariant over time.

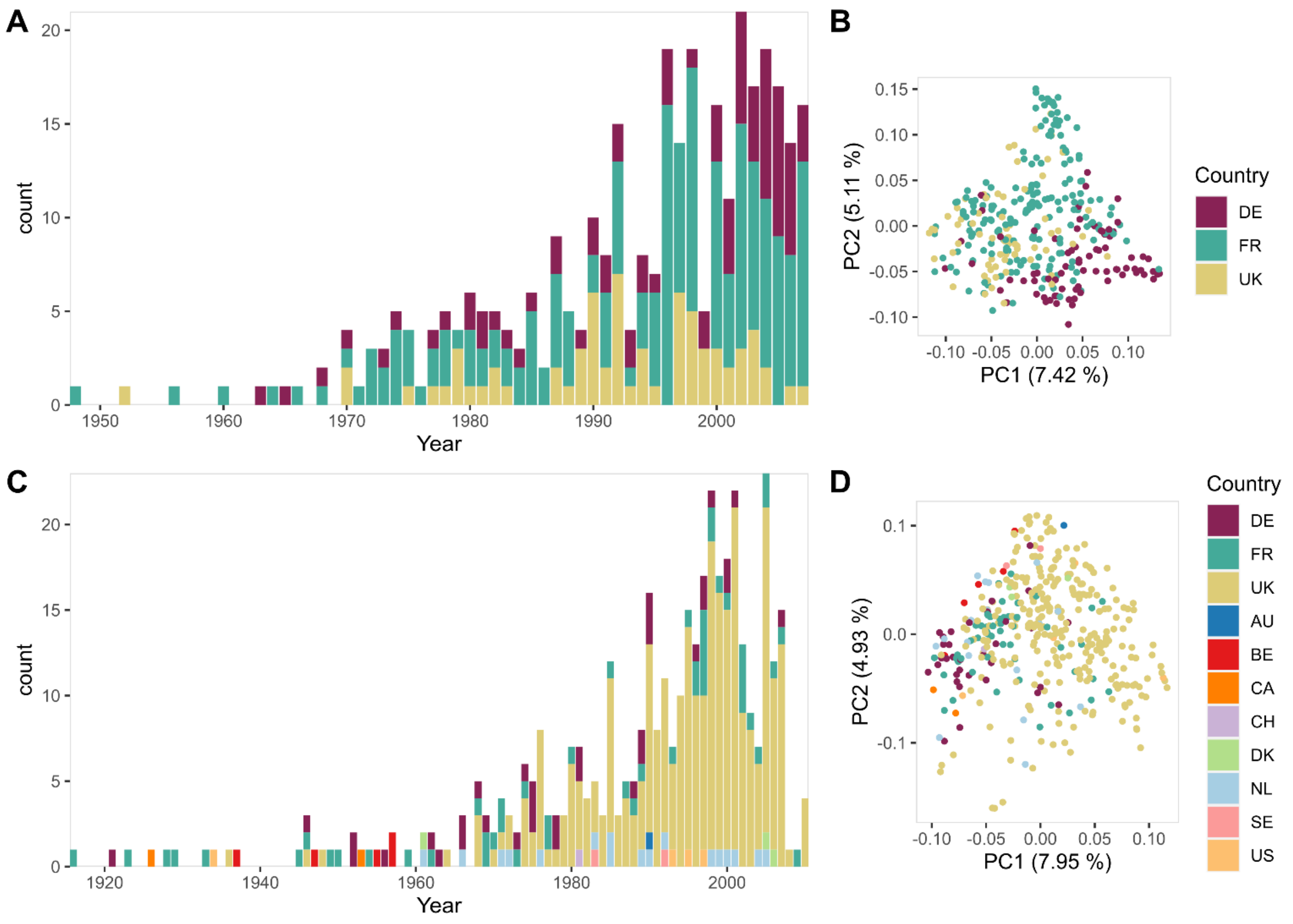

Figure S2. Distributions of 333 TG and 403 WAGTAIL winter wheat varieties according to years of release and countries of origin.

[**A**] Counts of TG varieties ranging from 1948 to 2007. [**B**] Plot of first two principal components using TG GBS dataset. [**C**] Counts of WAGTAIL varieties ranging from 1916 to 2010. [**D**] Plot of first two principal components using WAGTAIL dataset.

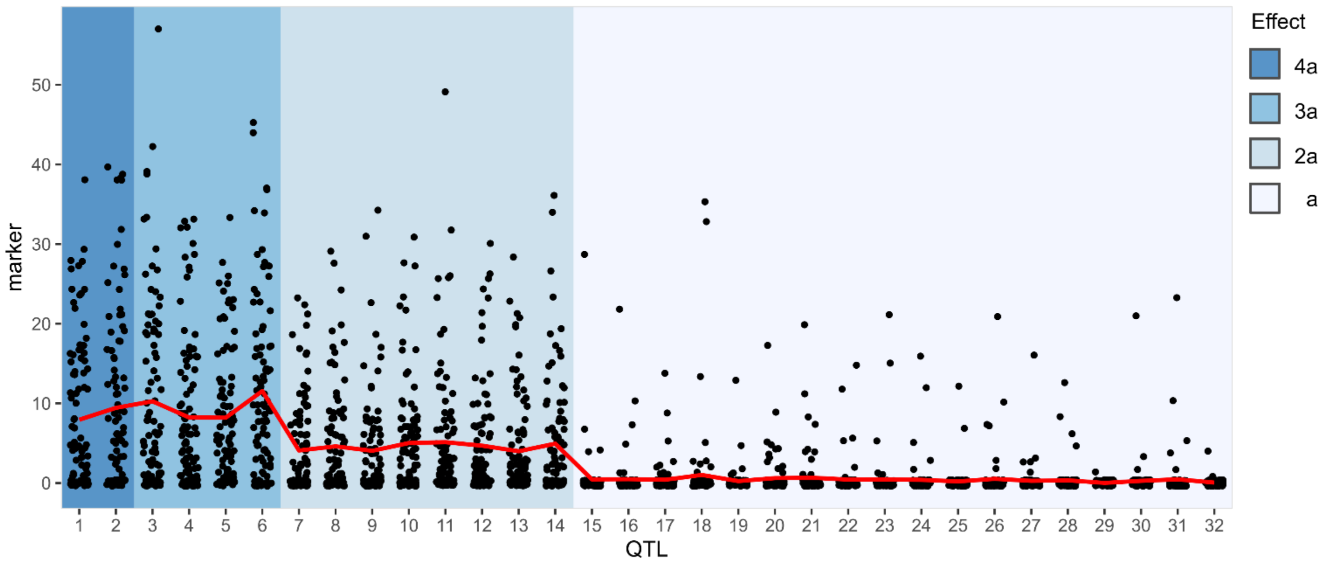

Figure S3. Number of significant markers across different simulated QTL frequencies and effects in the selected population.

The QTLs are simulated such that the initial frequencies are 1/32 (QTL-1,2), 2/32 (QTL-3,4), …, and 16/32 (QTL-31,32). Each point represents the number of significant markers within 5 cM of the QTL in a simulation. The red line shows the mean number of significant markers across all 32 QTLs. The number of significant markers drops as the QTL initial frequency increases and effect decreases.

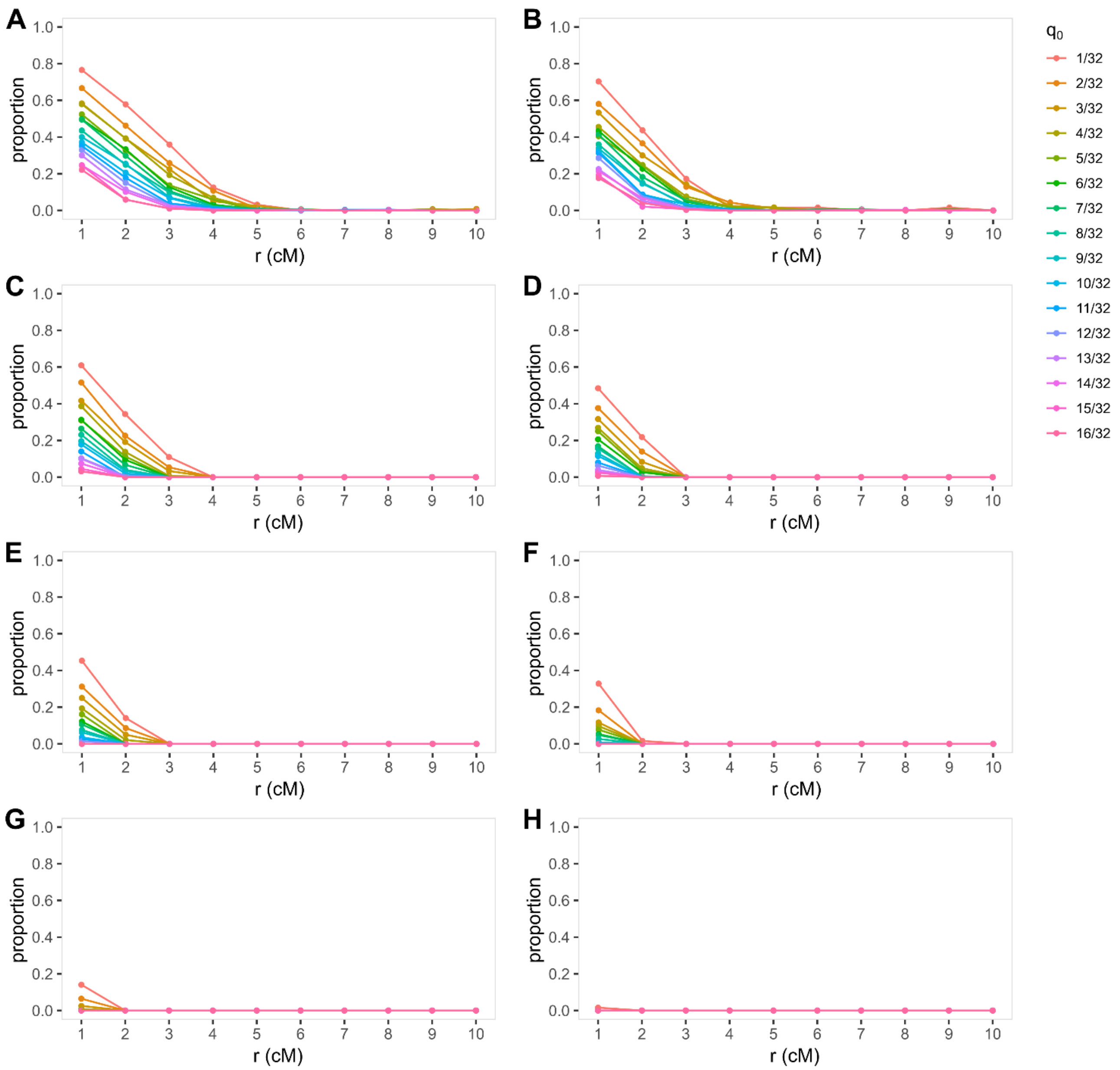

Figure S4. Detection limit of RALLY.

We tested the significances of proximal markers at distances of 1 to 10 cM away from a QTL under selection. All possible initial QTL-marker haplotypes were considered for each initial QTL allele frequency (q_0), and the proportions of haplotypes that led to significant markers were plotted. The QTL allele frequencies in [**A,C,E,G**] increase under a logistic model while the QTL allele frequencies in [**B,D,F,H**] increase under a linear model. The results are separated by markers that are [**A,B**] significant in at least 1 out of 100 simulations, [**C,D**] significant in at least 10 out of 100 simulations, [**E,F**] significant in at least 50 out of 100 simulations and [**G,H**] significant in all 100 simulations.

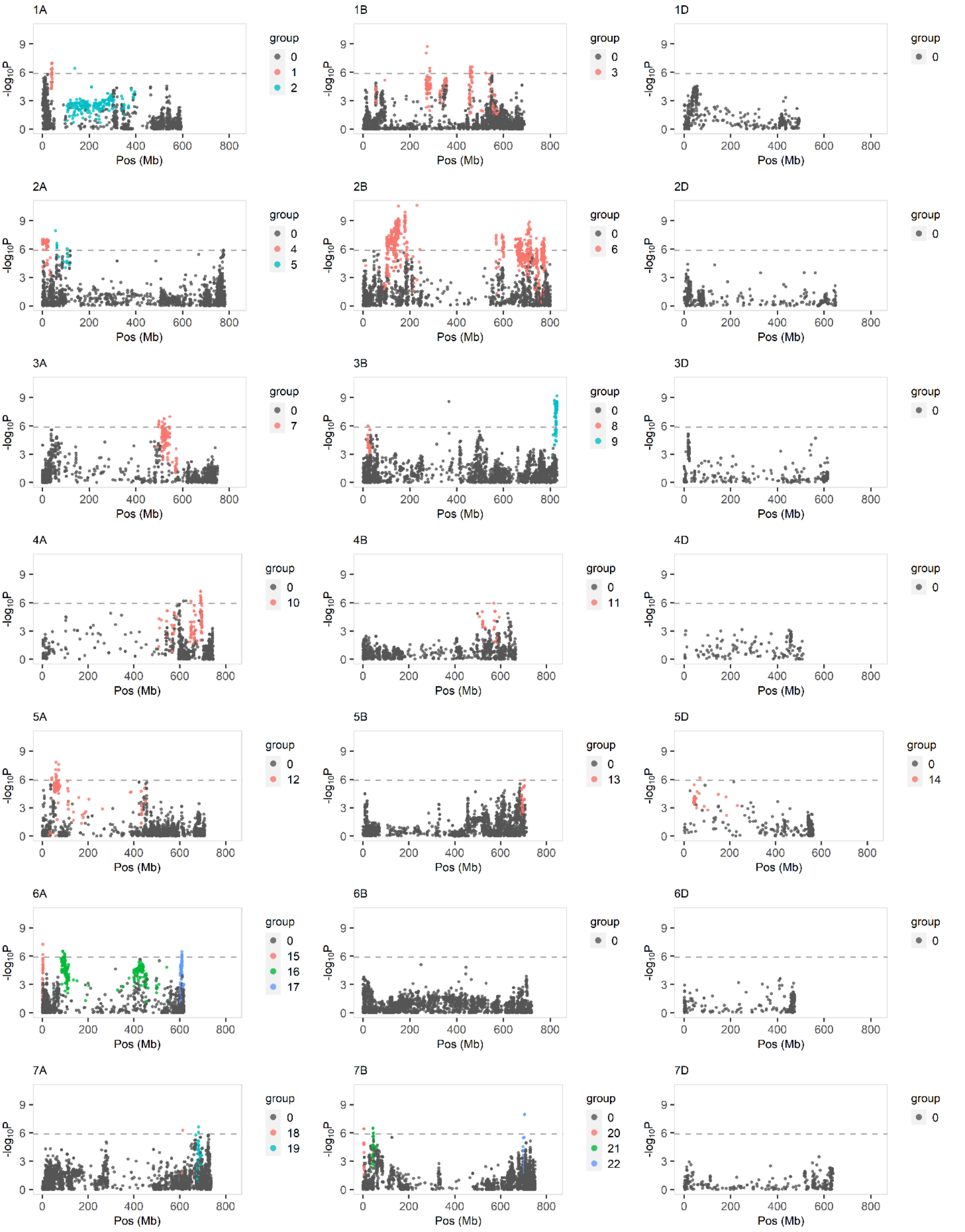

Figure S5. RALLY QSL groups in TG panel.

RALLY peaks and their linked markers ($r^2 > 0.2$) are grouped and highlighted. The dashed horizontal line represents the Bonferroni threshold of 0.05.

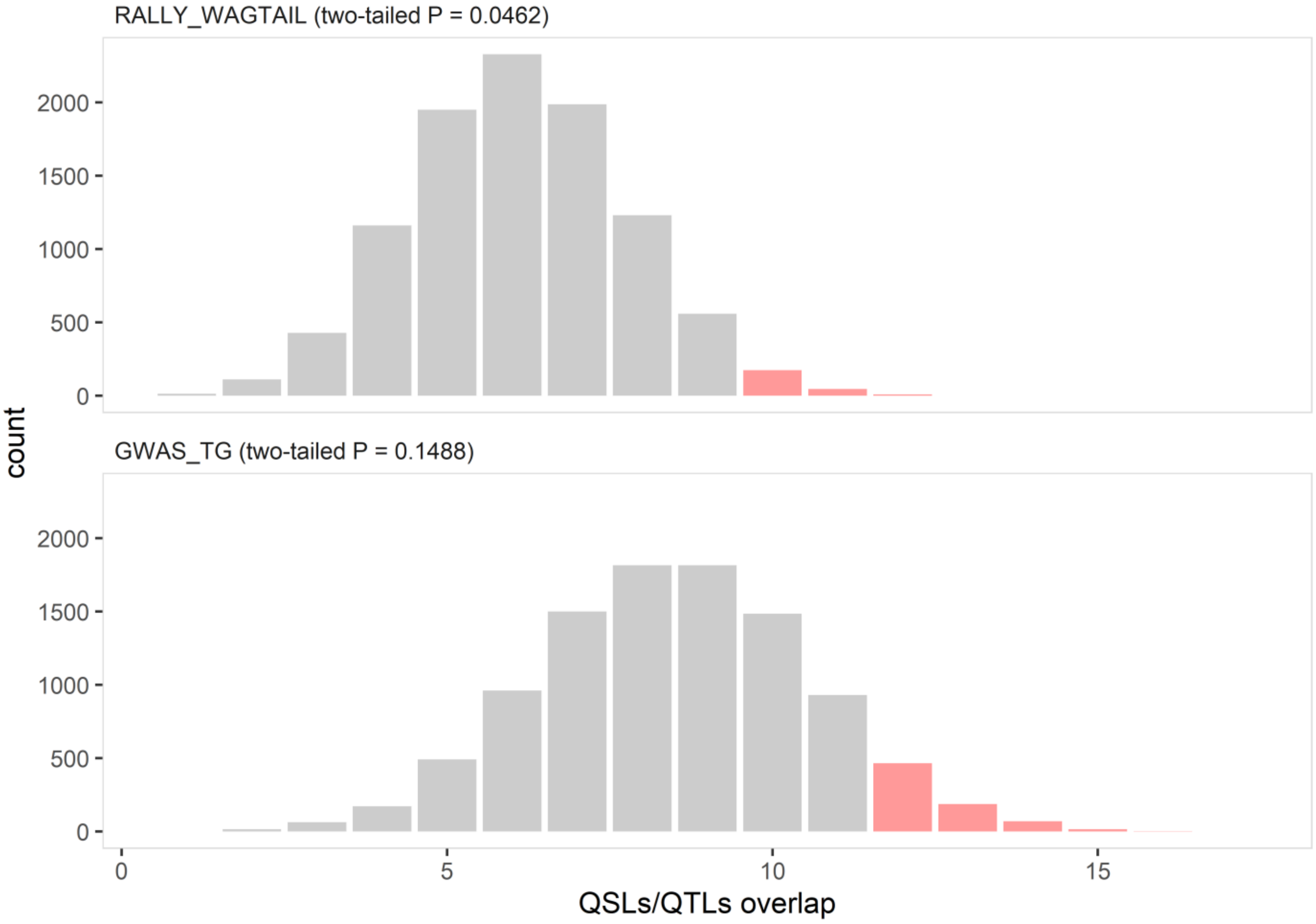

Figure S6. Randomization test results for QSLs/QTLs overlap.

We randomized the positions of 22 RALLY QSLs in the TG panel and calculated the overlap between these random QSLs and RALLY QSLs in the WAGTAIL panel (top), as well as between these random QSLs and GWAS QTLs in the TG panel (bottom). The same process was repeated for 10,000 randomizations. Histogram bars that equal or exceed the observed overlap (10 out 22 between RALLY in TG and WAGTAIL panels, 12 out of 22 between RALLY and GWAS in TG panels) are highlighted in red. The two-tailed P-values are calculated as twice the counts in the red bars divided by the total number of randomization.

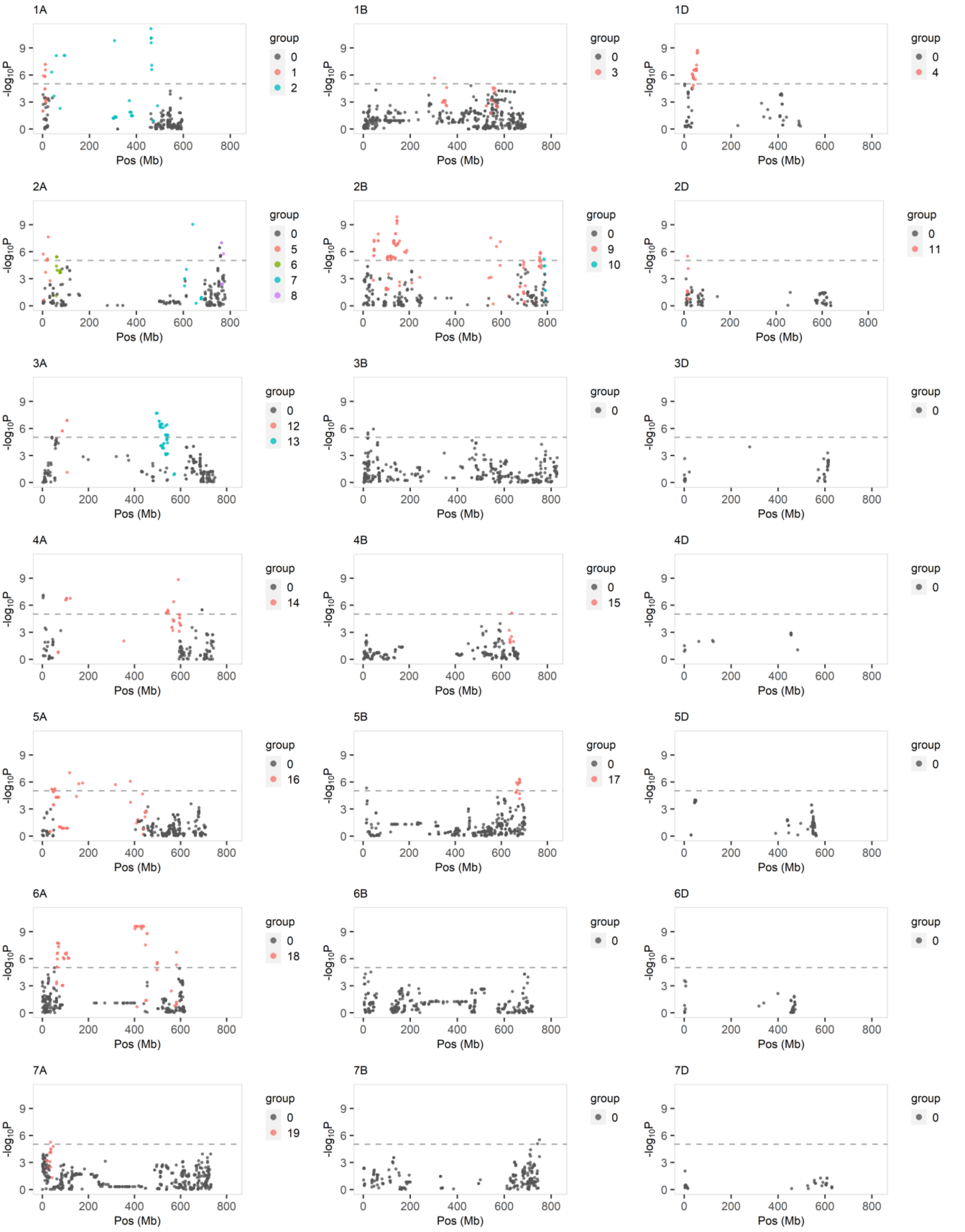

Figure S7. RALLY QSL groups in WAGTAIL panel.

RALLY peaks and their linked markers ($r^2 > 0.2$) are grouped and highlighted. The dashed horizontal line represents the Bonferroni threshold of 0.05.

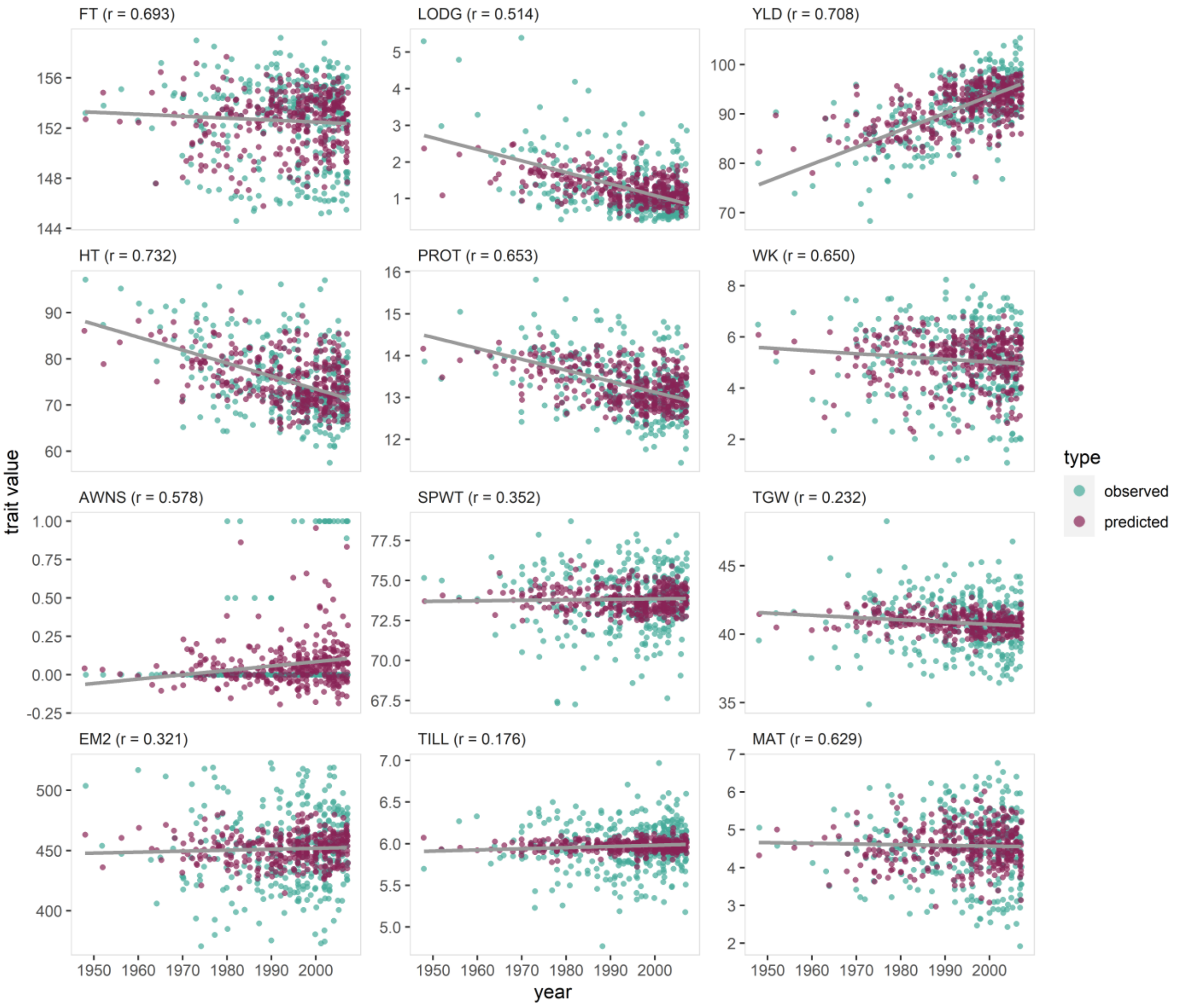

Figure S8. Predicted trait values using Ridge Regression (RR).

The traits are predicted using 5-fold cross validations (CVs). The predicted and observed trait values are plotted against the year of release. The correlations between predicted and observed trait values are shown at the top of each subplot. The gray linear regression line is fitted on the observed trait values to summarize the change in phenotypic trait values over the years.

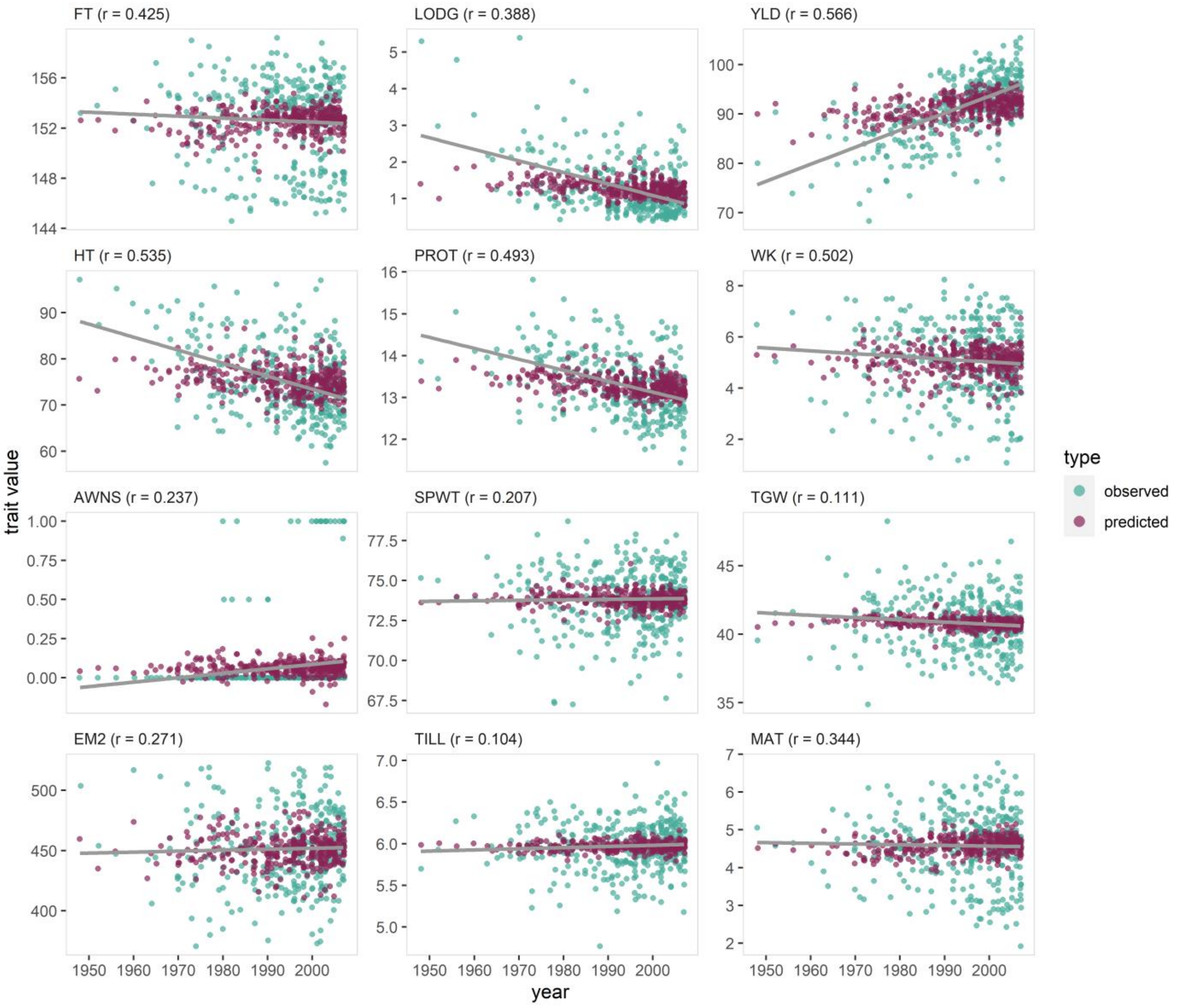

Figure S9 Predicted trait values using LASSO.

The traits are predicted using 5-fold cross validations (CVs). The predicted and observed trait values are plotted against the year of release. The correlations between predicted and observed trait values are shown at the top of each subplot. The gray linear regression line is fitted on the observed trait values to summarize the change in phenotypic trait values over the years.

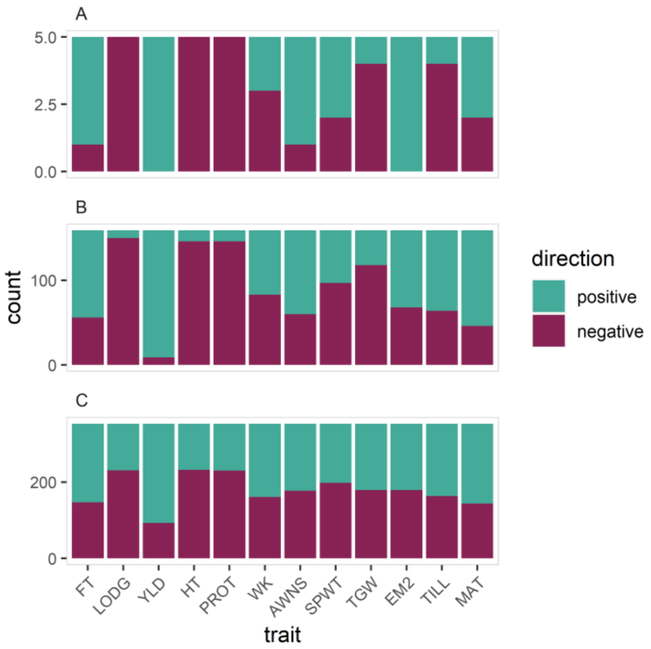

Figure S10. Distribution of the positive and negative LASSO effects in the increasing alleles.

[A] Markers with RALLY P-values of lower than the Bonferroni corrected threshold. [B] Markers with RALLY P-values between 0.05 and the Bonferroni corrected threshold. [C] Markers with RALLY P-values of higher than 0.05.

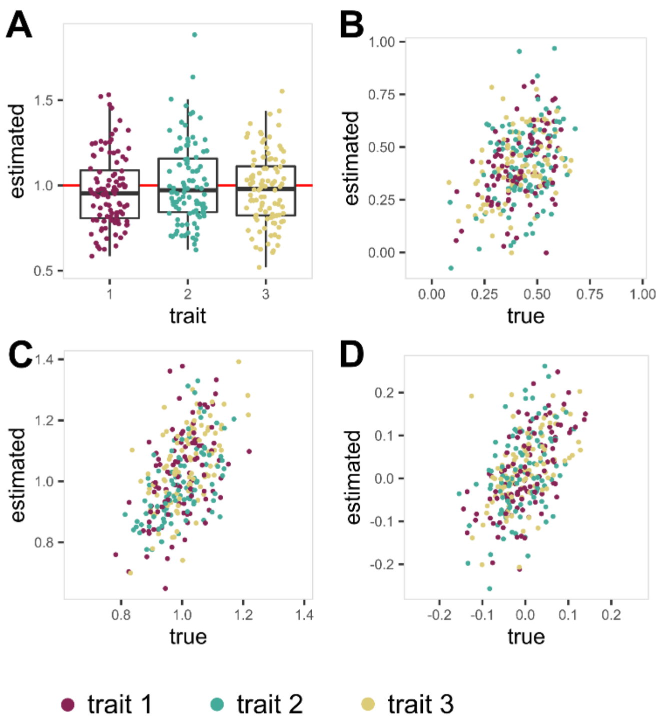

Figure S11. Genetic and residual variances-covariances in three simulated traits.

The estimated values from mixed linear model are shown in the Y-axis and the true simulated values are shown in the X-axis in [**B**-**D**]. Similar Y-axis is shown in [**A**] but since the true simulated genetic variances are always 1 (horizontal red line), the traits are shown in the X-axis instead. Each point represents a single simulation with 100 simulations in total. [**A**] Genetic variances, [**B**] Genetic covariances, [**C**] Residual variances, and [**D**] Residual covariances.

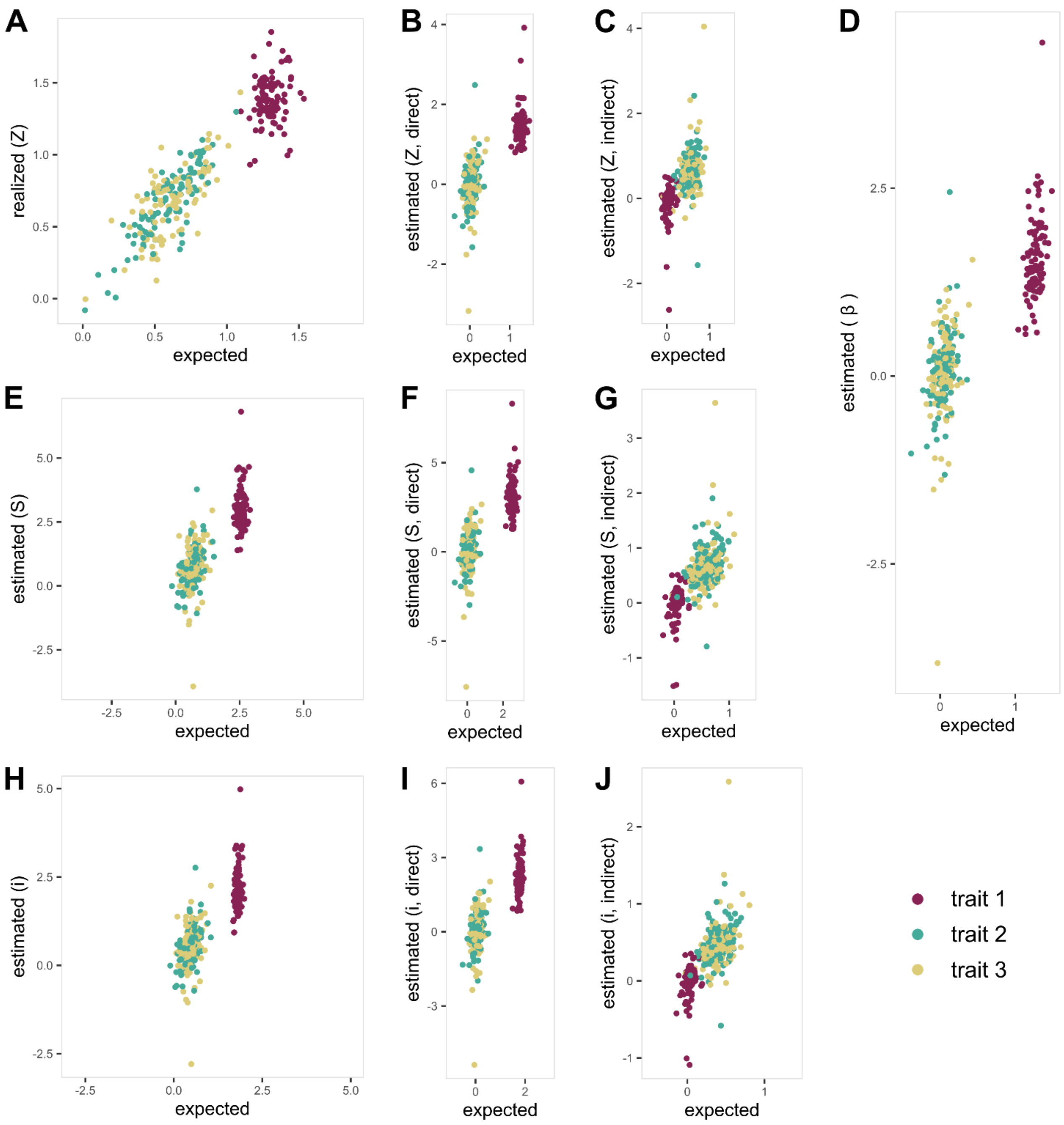

Figure S12. Estimated multivariate selection parameters in Sel1.

All estimated multivariate selection parameters are shown in the Y-axis and the true/expected values are shown in the X-axis. Note that selection responses are realized (*same as Figure S13A*) and do not depend on any estimated values from mixed linear model. [**A**] Selection responses, partitioned into [**B**] direct and [**C**] indirect. [**D**] Selection gradient. [**E**] Selection differential, partitioned into [**F**] direct and [**G**] indirect. [**H**] Selection intensity, partitioned into [**I**] direct and [**J**] indirect.

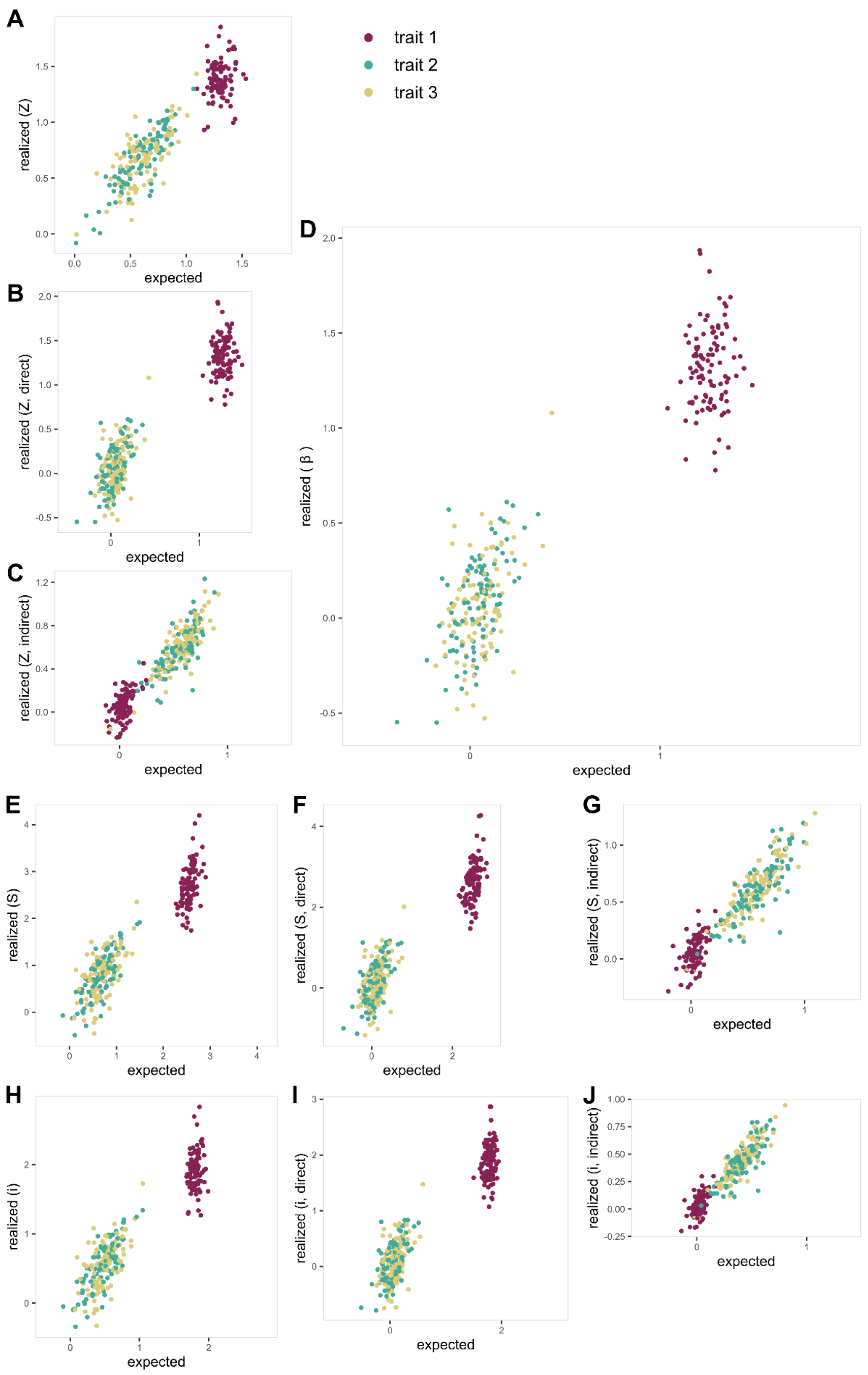

Figure S13. Realized multivariate selection parameters in Sel1.

All realized multivariate selection parameters are shown in the Y-axis and the true/expected values are shown in the X-axis. [**A**] Selection responses, partitioned into [**B**] direct and [**C**] indirect. [**D**] Selection gradient. [**E**] Selection differential, partitioned into [**F**] direct and [**G**] indirect. [**H**] Selection intensity, partitioned into [**I**] direct and [**J**] indirect.

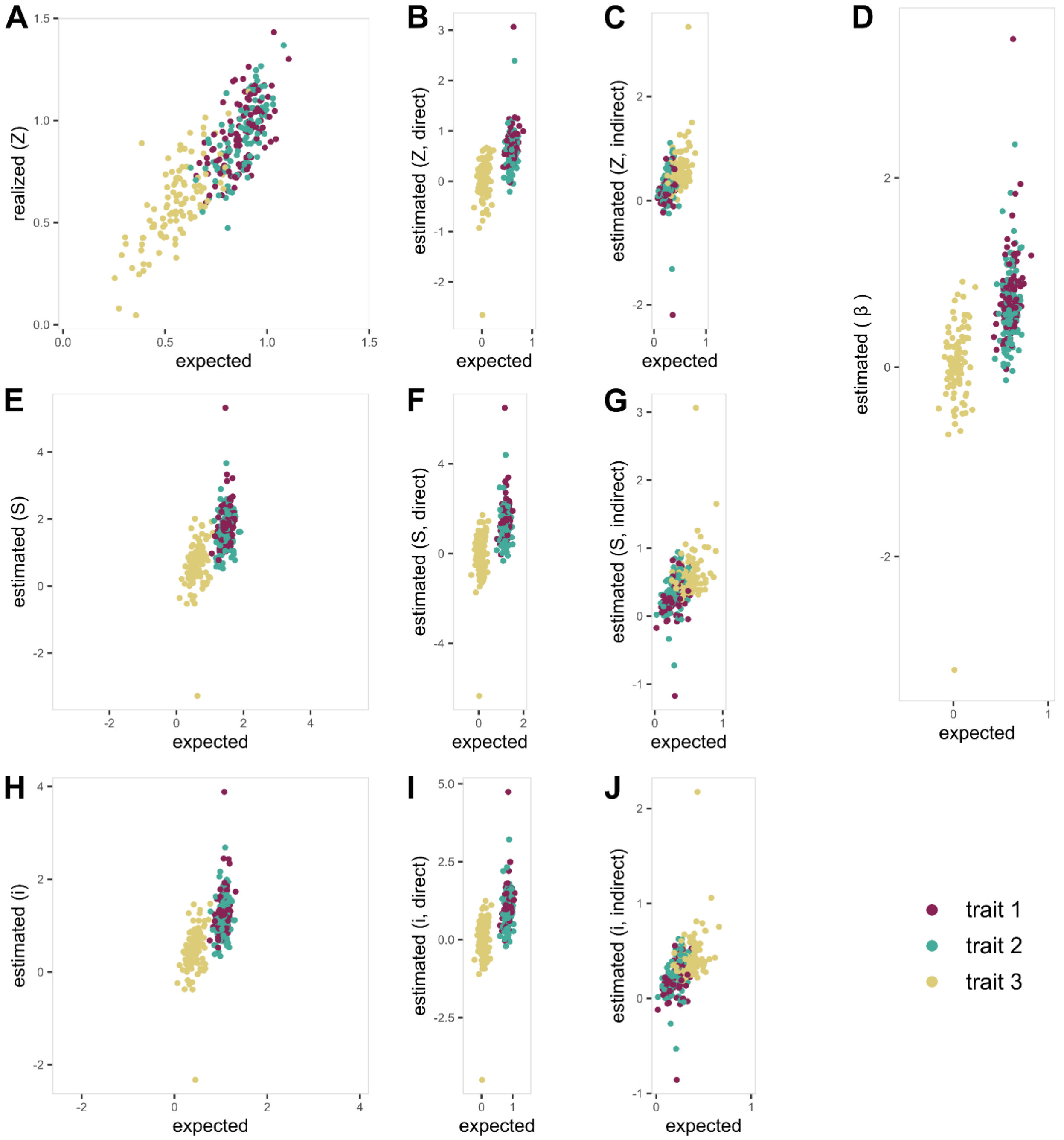

Figure S14. Estimated multivariate selection parameters in Sel2.

All estimated multivariate selection parameters are shown in the Y-axis and the true/expected values are shown in the X-axis. Note that selection responses are realized (*same as Figure S15A*) and do not depend on any estimated values from mixed linear model. [**A**] Selection responses, partitioned into [**B**] direct and [**C**] indirect. [**D**] Selection gradient. [**E**] Selection differential, partitioned into [**F**] direct and [**G**] indirect. [**H**] Selection intensity, partitioned into [**I**] direct and [**J**] indirect.

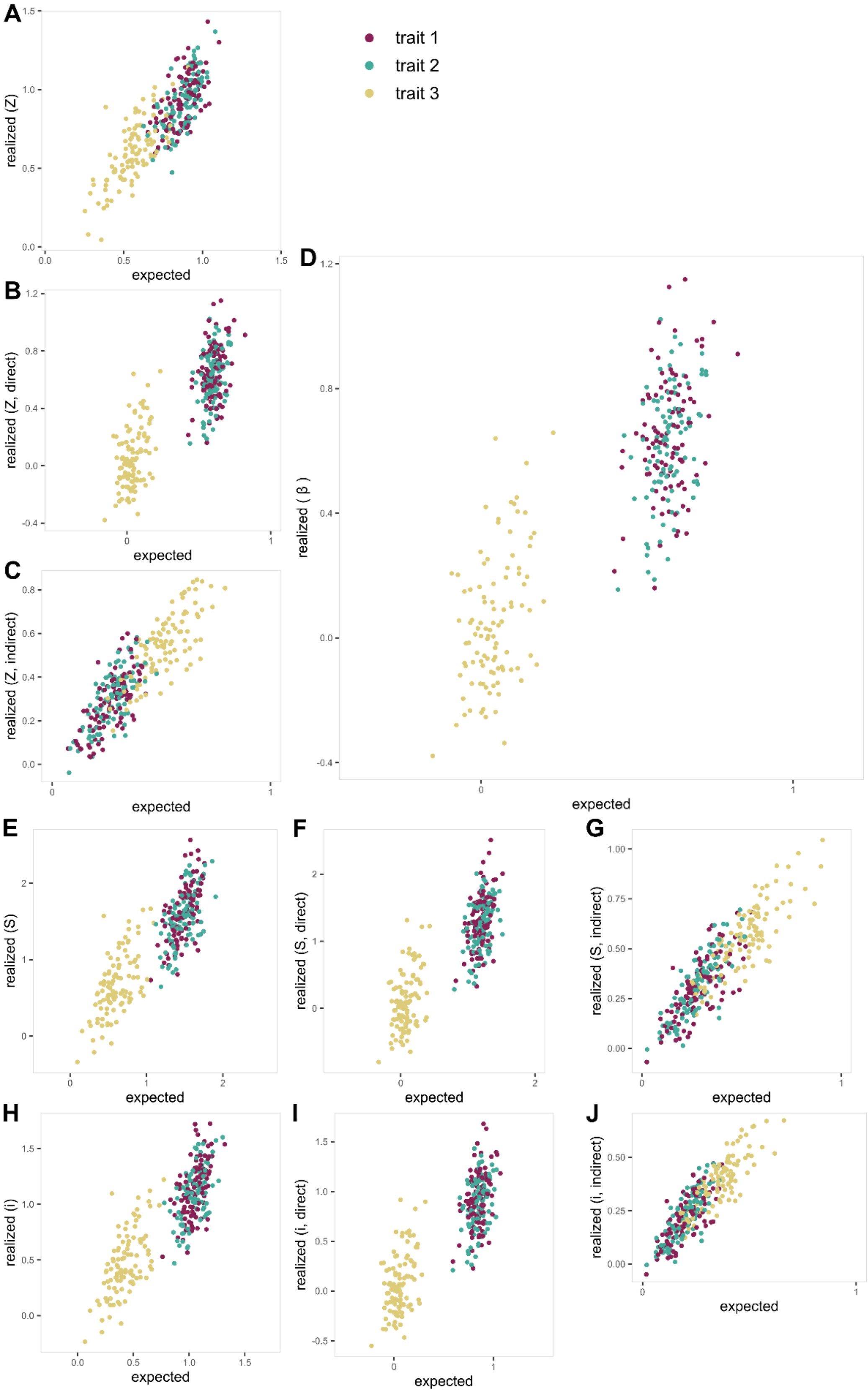

Figure S15. Realized multivariate selection parameters in Sel2.

All realized multivariate selection parameters are shown in the Y-axis and the true/expected values are shown in the X-axis. [**A**] Selection responses, partitioned into [**B**] direct and [**C**] indirect. [**D**] Selection gradient. [**E**] Selection differential, partitioned into [**F**] direct and [**G**] indirect. [**H**] Selection intensity, partitioned into [**I**] direct and [**J**] indirect.

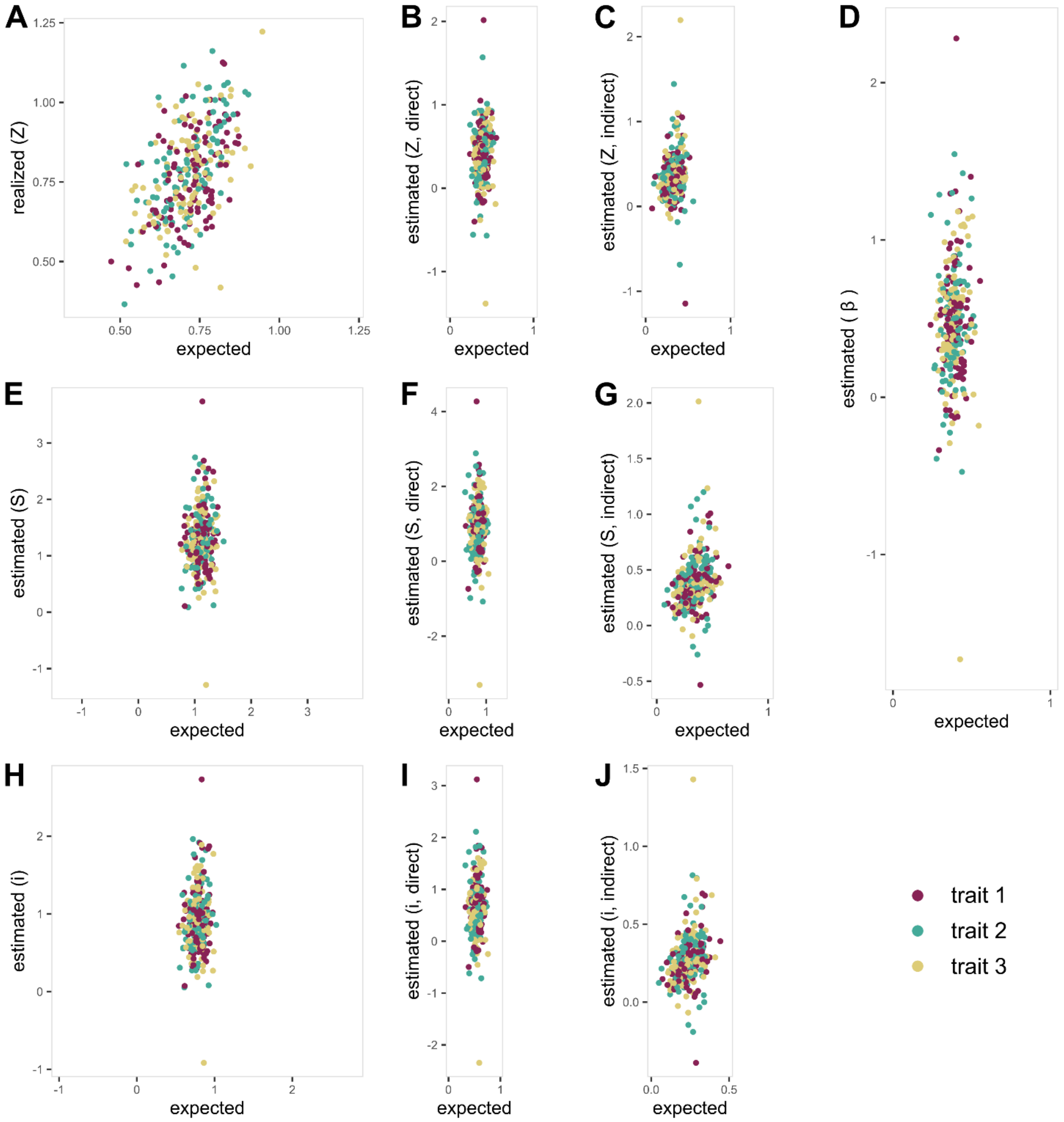

Figure S16. Estimated multivariate selection parameters in Sel3.

All estimated multivariate selection parameters are shown in the Y-axis and the true/expected values are shown in the X-axis. Note that selection responses are realized (*same as Figure S17A*) and do not depend on any estimated values from mixed linear model. [**A**] Selection responses, partitioned into [**B**] direct and [**C**] indirect. [**D**] Selection gradient. [**E**] Selection differential, partitioned into [**F**] direct and [**G**] indirect. [**H**] Selection intensity, partitioned into [**I**] direct and [**J**] indirect.

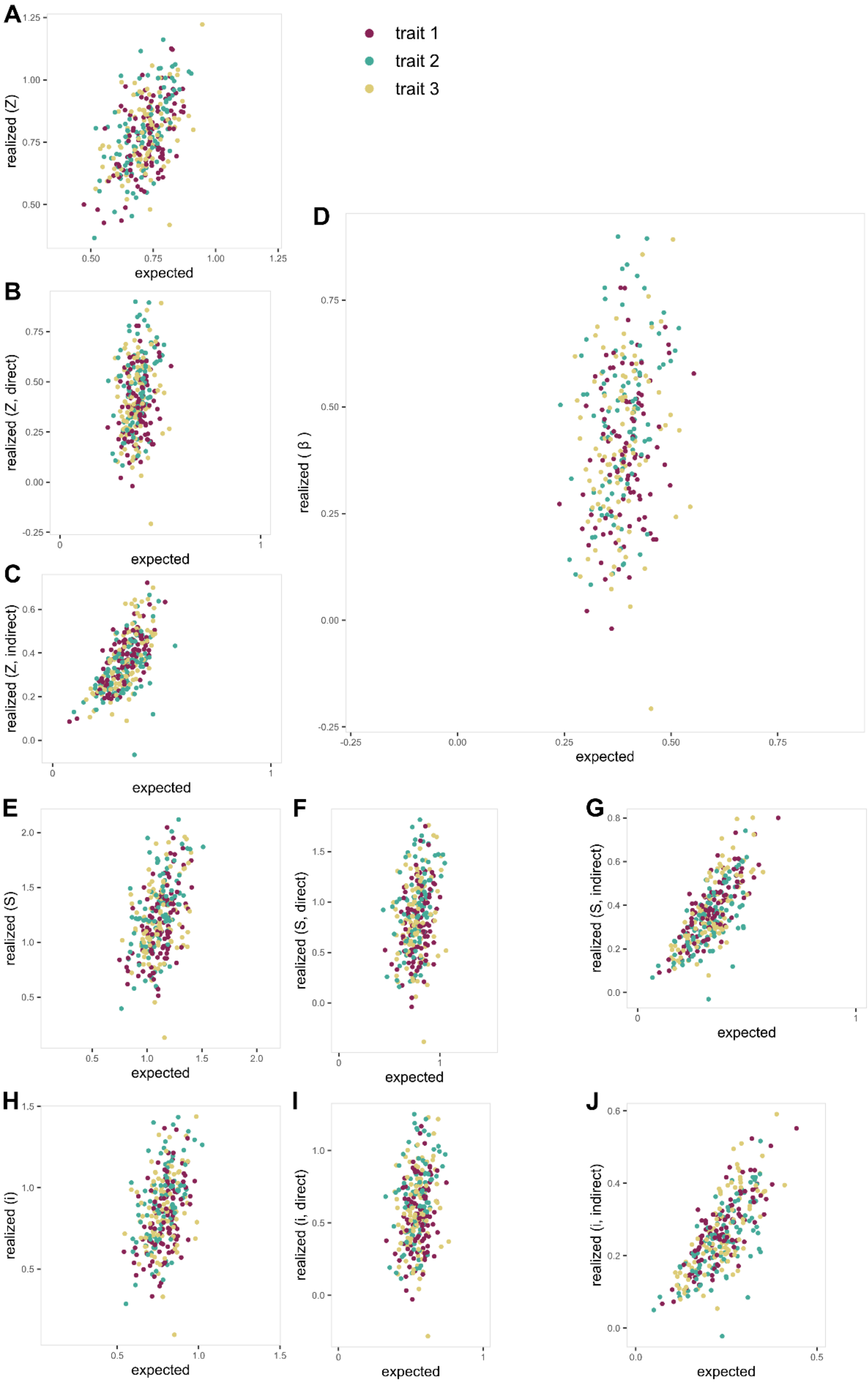

Figure S17. Realized multivariate selection parameters in Sel3.

All realized multivariate selection parameters are shown in the Y-axis and the true/expected values are shown in the X-axis. [**A**] Selection responses, partitioned into [**B**] direct and [**C**] indirect. [**D**] Selection gradient. [**E**] Selection differential, partitioned into [**F**] direct and [**G**] indirect. [**H**] Selection intensity, partitioned into [**I**] direct and [**J**] indirect.

References

1. Bentley AR, Scutari M, Gosman N, Faure S, Bedford F, Howell P, Cockram J, Rose GA, Barber T, Irigoyen J, Horsnell R, Pumfrey C, Winnie E, Schacht J, Beauchêne K, Praud S, Greenland A, Balding D, Mackay IJ (2014) Applying association mapping and genomic selection to the dissection of key traits in elite European wheat. Theor Appl Genet 127: 2619-2633.
2. Cheverud JM, Marroig G (2007) Comparing covariance matrices: random skewers method compared to the common principal components model. Genet Mol Biol 30: 461-469.
3. Covarrubias-Pazaran G (2016) Genome-assisted prediction of quantitative traits using the R package *sommer*. PLoS One 11: e0156744.
4. Falconer DS, Mackay TFC (1996) Introduction to quantitative genetics. Prentice Hall, Essex, England.
5. Gaynor RC, Gorjanc G, Hickey JM (2021) AlphaSimR: an R package for breeding program simulations. G3: Genes, Genomes, Genetics 11: jkaa017.
6. Ladejobi O, Mackay IJ, Poland J, Praud S, Hibberd JM, Bentley AR (2019) Reference genome anchoring of high-density markers for association mapping and genomic prediction in European winter wheat. Front Plant Sci 10: 1278.
7. Lande R, Arnold SJ (1983) The measurement of selection on correlated characters. Evolution 37: 1210-1226.
8. Mantel N (1967) The detection of disease clustering and a generalized regression approach. Cancer Res 27: 209-220.
9. Paradis E, Claude J, Strimmer K (2004) APE: analyses of phylogenetics and evolution in R language. Bioinformatics 20: 289-290.
10. R Core Team (2021) R: A language and environment for statistical computing. R Foundation for Statistical Computing, Vienna.
11. Revell LJ (2011) phytools: an R package for phylogenetic comparative biology (and other things). Methods Ecol Evol 3: 217-223.
12. Santantonio N, Jannink J-L, Sorrells M (2019) A low resolution epistasis mapping approach to identify chromosome arm interactions in allohexaploid wheat. G3: Genes, Genomes, Genetics 9: 675-684.
13. Yang Y, Aduragbemi A, Wei D, Chai Y, Zheng J, Qiao P, Cui C, Lu S, Chen L, Hu Y-G (2021) Large-scale integration of meta-QTL and genome-wide association study discovers the genomic regions and candidate genes for yield and yield-related traits in bread wheat. Theor Appl Genet 134: 3083-3109.
